# Climate and human water use diminish wetland networks supporting continental waterbird migration

**DOI:** 10.1111/gcb.15010

**Published:** 2020-02-13

**Authors:** J. Patrick Donnelly, Sammy L. King, Nicholas L. Silverman, Daniel P. Collins, Eduardo M. Carrera‐Gonzalez, Alberto Lafón‐Terrazas, Johnnie N. Moore

**Affiliations:** ^1^ Intermountain West Joint Venture – U.S. Fish and Wildlife Service Missoula MT USA; ^2^ U.S. Geological Survey Louisiana Cooperative Fish and Wildlife Research Unit 124 School of Renewable Natural Resources Louisiana State University Baton Rouge LA USA; ^3^ Adaptive Hydrology LLC Missoula MT USA; ^4^ U.S. Fish and Wildlife Service Region 2 Migratory Bird Office Albuquerque NM USA; ^5^ Ducks Unlimited de Mexico San Pedro Garza Garcia Mexico; ^6^ PROFAUNA A.C. Chihuahua City Mexico; ^7^ Group For Quantitative Study of Snow and Ice Department of Geosciences University of Montana Missoula MT USA

**Keywords:** agricultural irrigation, climate, endorheic lake and wetland desiccation, flyway connectivity, Mexico, migratory waterbirds, North America, wetland ecosystem collapse

## Abstract

Migrating waterbirds moving between upper and lower latitudinal breeding and wintering grounds rely on a limited network of endorheic lakes and wetlands when crossing arid continental interiors. Recent drying of global endorheic water stores raises concerns over deteriorating migratory pathways, yet few studies have considered these effects at the scale of continental flyways. Here, we investigate the resiliency of waterbird migration networks across western North America by reconstructing long‐term patterns (1984–2018) of terminal lake and wetland surface water area in 26 endorheic watersheds. Findings were partitioned regionally by snowmelt‐ and monsoon‐driven hydrologies and combined with climate and human water‐use data to determine their importance in predicting surface water trends. Nonlinear patterns of lake and wetland drying were apparent along latitudinal flyway gradients. Pervasive surface water declines were prevalent in northern snowmelt watersheds (lakes −27%, wetlands −47%) while largely stable in monsoonal watersheds to the south (lakes −13%, wetlands +8%). Monsoonal watersheds represented a smaller proportion of total lake and wetland area, but their distribution and frequency of change within highly arid regions of the continental flyway increased their value to migratory waterbirds. Irrigated agriculture and increasing evaporative demands were the most important drivers of surface water declines. Underlying agricultural and wetland relationships however were more complex. Approximately 7% of irrigated lands linked to flood irrigation and water storage practices supported 61% of all wetland inundation in snowmelt watersheds. In monsoonal watersheds, small earthen dams, meant to capture surface runoff for livestock watering, were a major component of wetland resources (67%) that supported networks of isolated wetlands surrounding endorheic lakes. Ecological trends and human impacts identified herein underscore the importance of assessing flyway‐scale change as our model depictions likely reflect new and emerging bottlenecks to continental migration.

## Graphical abstract

Migrating waterbirds moving between upper and lower latitudinal breeding and wintering grounds rely on a limited network of endorheic (hydrologically landlocked) lakes and wetlands when crossing arid continental interiors. We document strengthening patterns of landscape desiccation representing emerging bottlenecks to migratory pathways in western North America. Lake (a) and wetland (b) drying were pervasive in numerous watersheds, driven by agricultural water use and rising evaporative demands implying a greater need for focused wetland and waterbird conservation in this expansive region. Sustainability of waterbird migration networks will require adaptive changes to flyway conservation priorities considerate of accelerating lake and wetlands loss.

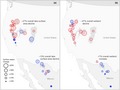

## 1 INTRODUCTION

Water‐limited ecosystems account for 40% of terrestrial land surfaces globally and support upwards of 2 billion people (Gilbert, 2011). Nearly half of these arid and semi‐arid regions are made up of endorheic watersheds in which all runoff converges in terminal water bodies topographically landlocked from the ocean (Wada et al., 2011). Water scarcity places vital ecological and economic importance on endorheic watersheds as they are often associated with sizable lakes and wetland systems in otherwise arid landscapes. Endorheic water stores are driven predominantly by precipitation inputs and groundwater exchange that equilibrate through evaporation. Recent declines in global endorheic water storage (Wang et al., 2018) suggest increased evaporative demands, due to warming temperatures. More frequent droughts are threatening this delicate ecosystem water balance as growing human populations (Wada, van Beek, Wanders, & Bierkens, 2013) and intensifying climate change (Dai, 2013) are increasing water consumption and accelerating endorheic withdrawals (Wurtsbaugh et al., 2017).

Waterbirds crossing arid continental interiors concentrate in a limited number of important wetland sites during migration (Haig, Mehlman, & Oring, 1998; Morrison & Myers, 1989). These wetlands are part of larger flyway networks supporting global migration of waterbirds that synchronize movements and stopover sites to meet annual lifecycle demands as they travel between upper and lower latitudinal breeding and wintering grounds (Boere & Stroud, 2006). Endorheic/terminal lakes and wetlands, hereafter ‘lakes’ and ‘wetlands’, are key links in continental flyways with numerous sites designated as critically important to waterbird populations (Frazier, 1999; Kushlan et al., 2002; NAWMP, Canadian Wildlife Service, U.S. Fish and Wildlife Service, & Ambiente y Recursos Naturales, 2012; Senner, Andres, & Gates, 2016). Global drying of endorheic watersheds (Wang et al., 2018) raises concerns over the maintenance of flyway connectivity in arid and semi‐arid regions. Reliance on a small number of important migratory stopovers make some waterbird populations vulnerable to landscape change as loss of individual wetlands can dramatically alter resource abundance and distribution (Roshier, Robertson, Kingsford, & Green, 2001; Wilsey, Taylor, Stockdale, & Stockdale, 2017), that can affect energetic cost of migration (Buehler & Piersma, 2008; Devries, Brook, Howerter, & Anderson, 2008). Dewatering of endorheic watersheds has the potential to impact long‐term population dynamics as carry‐over effects driven by deteriorating migratory habitats reduce waterbird survivorship in subsequent life‐history events (Hua, Tan, Chen, & Ma, 2015; Sedinger & Alisauskas, 2014).

Arid and semi‐arid mid‐latitudes of western North America are among the most important inland waterbird flyways in the Western Hemisphere (Oring & Reed, 1997; Wilsey et al., 2017). In this region, migratory pathways are structured around endorheic watersheds that support large saline and freshwater lakes in addition to freshwater palustrine wetlands occurring along lake peripheries and throughout surrounding riparian drainages. Close proximity of these distinctive wetland environments (lacustrine, palustrine, freshwater, and saline) concentrates biodiversity within endorheic watersheds. These habitats collectively make up a wetland network supporting 94 migratory waterbird species and millions of individual birds during breeding, wintering, and migration (Drewien, Brown, & Benning, 1996; Drewien, Terrazas, Taylor, Barraza, & Shea, 2003; Jehl, 1994; Oring, Neel, & Oring, 2000; Oring & Reed, 1997; Paul & Manning, 2002). Continental waterbird populations reliant on these habitats include the following: 70% of waterfowl in western North America (IWJV, 2013), 99% of eared grebes (*Podiceps nigricollis*), 90% of Wilson's Phalaropes (*Phalaropus tricolor*), 50% of American Avocets (*Recurvirostra americana*), 50% of American White Pelicans (*Pelecanus erythrorhynchos*), and 50% of Western Snowy Plovers (*Charadrius nivosus nivosus*; Ellis & Jehl, 2003; Oring et al., 2000; Paul & Manning, 2002).

**Figure 1:**
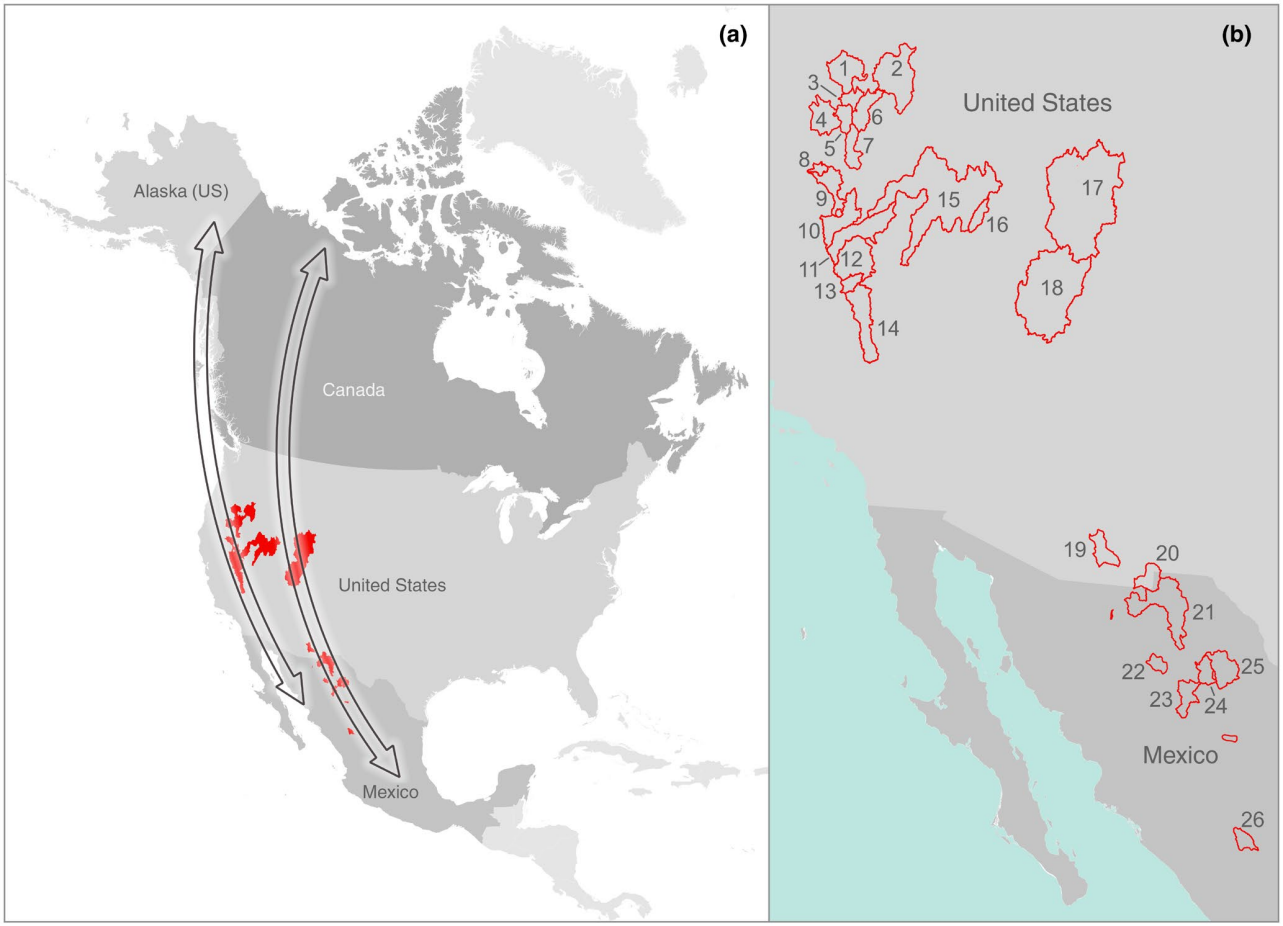
Endorheic lake watersheds in the context of generalized migratory waterbird flyways in western North America (a). Watersheds are partitioned by snowmelt‐ (1–18) and monsoon‐driven (19–26) hydrologies (b). Watersheds include (1) Summer Lake, (2) Harney Basin, (3) Lake Abert, (4) Tule Lake Basin, (5) Goose Lake, (6) Warner Valley, (7) Alkali Lakes, (8) Eagle Lake, (9) Honey Lake, (10) Pyramid Lake, (11) Carson Sink, (12) Walker Lake, (13) Mono Lake, (14) Owens Lake, (15) Humboldt Sink, (16) Ruby Valley, (17) Great Salt Lake, (18) Sevier Lake, (19) Willcox Playa, (20) Castillo Playa, (21) Ascensión, (22) Laguna de Babicora, (23) Laguna de Bustillos, (24) Laguna de Ojo, (25) Laguna de Cuervo, and (26) Laguna de Santiaguillo

Endorheic watersheds offer a unique framework to measure flyway resiliency due to their closed basin hydrology that act as landscape‐scale monitors of ecosystem water balance. Changes to lake and wetland area can be isolated to directly assess sensitivity to climate and human water use. To quantify flyway‐scale resilience, we used 35 years (1984–2018) of satellite imagery to reconstruct annual surface water area/extent in 26 North American endorheic watersheds (Figure 1). These watersheds have been designated as regionally, internationally, hemispherically, or globally important to waterbird populations (Table 1). To better understand resilience of migratory flyways, we combined surface water data with climate and human water‐use factors to determine their importance in predicting spatiotemporal patterns of lake and wetland area trends. The analysis provides an ecological perspective to changing flyway conditions at a continental scale that to this point have remained largely unexplored. Study outcomes give new insight to support development of regionally specific wetland conservation strategies to offset emerging migratory bottlenecks. Although concentrated in North America, this research has application to all eight global waterbird flyways (Wetlands International, 2012), all of which cross arid regions.

**Table 1:**
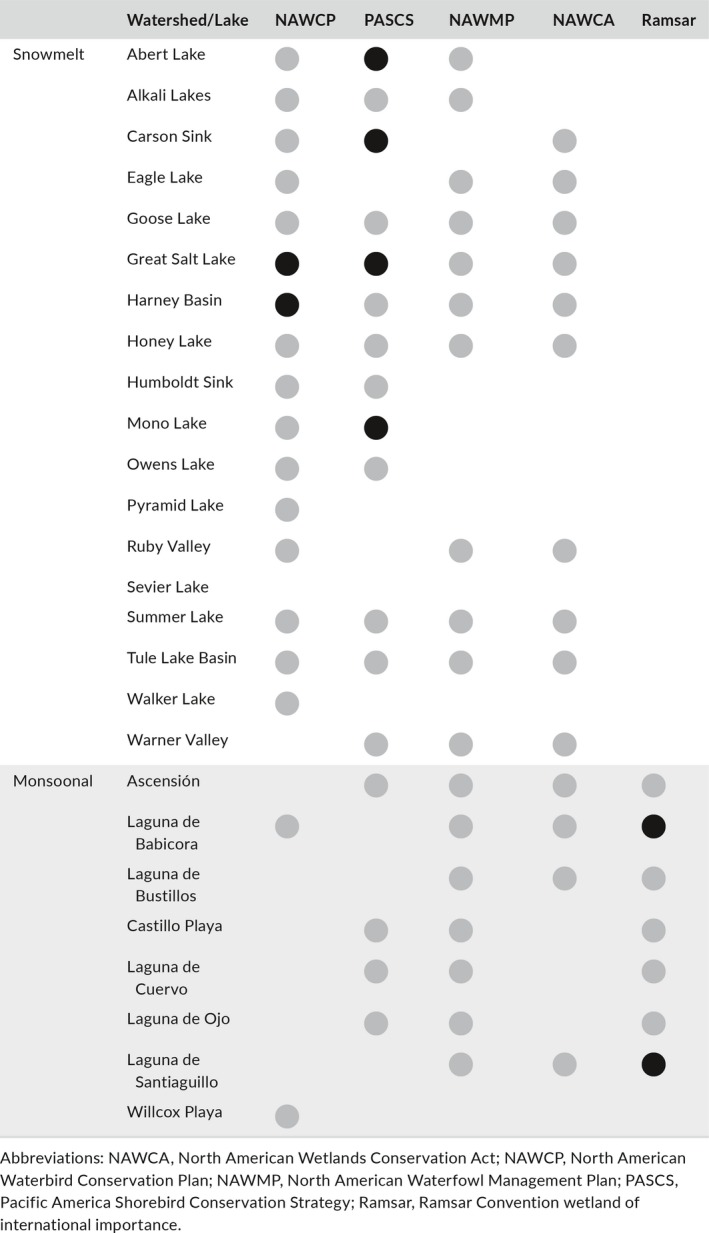
Alignment of North America's designated important waterbird habitats and endorheic watersheds. Markers identify recognition or investment made by continental waterbird plans or wetland conservation initiatives. Black indicates elevated global, hemispheric, or international importance. Ramsar sites contain designated (black) and proposed (gray) status

## 2 METHODS

### 2.1 Study area

Our study area encompassed endorheic watersheds in arid, and semi‐arid regions of North America that makeup a network of important lakes and surrounding wetland habitats utilized by migratory waterbirds in western flyways of the continent (Lincoln, 1935; Figure 1; Table 1). Watersheds were partitioned by dominance of snowmelt‐ or monsoon‐driven hydrologic regimes to isolate flyway resilience to changing climate and human water use along latitudinal gradients.

The snowmelt region encompassed 18 northern endorheic watersheds, hereafter ‘snowmelt watersheds’, in the Great Basin of the United States that receive a majority of annual precipitation as accumulating snowpack from winter (November–February) storm front passage (Fyfe et al., 2017; Klos, Link, & Abatzoglou, 2014). Snowpack accounts for 50%–70% of spring runoff that feed perennial stream flows supporting saline and freshwater lakes and a mosaic of emergent freshwater seasonally and semi‐permanently flooded wetlands.

The monsoon region encompassed eight southern endorheic watersheds in the northern highlands of Mexico and the southwestern United States, hereafter ‘monsoonal watersheds’ (Figure 1; Table 1). These watersheds receive their precipitation primarily during warm summer months (July–September) from convective thunderstorms influenced by complex interactions of subtropical ocean moisture and continental land masses (Adams & Comrie, 1997). Intermittent surface water flows from storm runoff support freshwater lakes and a limited wetland abundance. The Ascensión region in Mexico encompasses a single monsoonal watershed made up of multiple large interconnected lakes that included Laguna de Guzmán, Laguna de San Juan, and Laguna de Santa Mariá.

### 2.2 Estimating surface water trends

Lake and wetland surface water area was monitored annually from 1984 to 2018 using Landsat 5 Thematic Mapper (1984–2011) and Landsat 8 Operational Land Imager (2013–2018) satellite imagery. A gap in satellite coverage prevented surface water monitoring in 2012. Following an approach outlined by Donnelly et al. (2019), surface water area was measured using constrained spectral mixture analysis (SMA; Adams & Gillespie, 2006) that allowed proportional estimations of water contained within a continuous 30 × 30 m pixel grid (Halabisky, Moskal, Gillespie, & Hannam, 2016; Jin, Lang, Yeo, Stehman, & Stephen, 2017). This approach provides an accurate account of surface water area/extent when detectability is reduced due to interspersion of emergent vegetation, shallow, or turbid water (DeVries et al., 2017), characteristics common to lakes and wetlands in arid and semi‐arid regions (Jolly, McEwan, & Holland, 2008). Areas containing cloud, cloud shadow, snow, and ice were masked using the Landsat CFMask band (Foga et al., 2017). All unmasked pixels in Landsat 30 m visible, near‐infrared, and short wave infrared bands were incorporated into the SMA with the exception of Landsat 8 coastal aerosol band.

Training data for SMA were extracted from satellite imagery as spectral endmembers unique to individual images classified. Training site locations were representative of homogeneous land cover types mapped as water, wetland vegetation, upland, and alkali soil. Spectral endmembers for water were collected using image masks generated from 99th percentile normalized difference water index values (McFeeters, 1996). Mask extents were coincident with large deep water lakes within or proximal to endorheic watersheds. A similar masking approach was applied to collect wetland vegetation endmembers using normalized difference vegetation indices (Box, Holben, & Kalb, 1989). Sampling was constrained to sites coincident with seasonally flooded wetlands and were representative of associated plant phenology. SMA requires minimal training data (Adams & Gillespie, 2006) which allowed upland and alkali soil endmembers to be generated from a small number of static plots within endorheic watersheds (*n* = 2; 0.5–1 km^2^). Upland plots were associated with homogenous shrublands characterized by low vegetative productivity and high soil exposure. Alkali soil plots were coincident with dry lake basins in areas of surface mineral deposits. Plot locations were identified using high‐resolution (<0.5 m) multispectral satellite imagery or field reconnaissance.

Surface water extent within endorheic watersheds was averaged annually within offset 6 months of seasonal periods for the snowmelt (April–September) and monsoonal (October–March) regions (see Figure 1). Periods aligned broadly with known spatiotemporal waterbird breeding, migration, and wintering patterns (Baldassarre, 2014; Kushlan et al., 2002; Senner et al., 2016). Satellite data within annual periods of 6 months were averaged into single multi‐spectral images and classified using SMA to produce seasonal estimates of lake and wetland extent from 1984 to 2018; 2012 omitted. Applying this approach made it possible to measure ecological variability influencing migratory waterbird habitat and simultaneously reduced the potential of monitoring gaps in Landsat data caused by clouds and cloud shadows. Lake and wetland areas were assumed to represent broader ecological trends in habitat availability; however, we acknowledge intra‐seasonal variance within periods monitored can also influence resource values for waterbirds (González‐Gajardo, Sepúlveda, & Schlatter, 2009).

Annual surface water estimations were clipped and summarized spatially within digitized lake and wetland polygons. This process minimized the potential of false water positives by removing anthropogenic features (e.g. buildings and asphalt) and topographic shadow known to be misclassified as water when using SMA (DeVries et al., 2017). Polygons were classified into functional groups to filter water bodies (e.g. salt evaporation ponds, large reservoirs, and livestock ponds) extraneous to endorheic lake and wetland trends. Because emergent vegetation or high turbidity could partially mask estimated proportion of pixel areas covered with water (Donnelly et al., 2019), we considered pixels fully inundated when water was present. Pixels containing <10% surface water were omitted from summaries to minimize over estimation of surface water area.

Final analyses resulted in an annual 35 year time‐series estimate (1984–2018) of lake and wetland surface water area within endorheic watersheds. Freshwater wetlands occurring along the periphery of saline lakes as a result of groundwater discharge from springs or runoff from streams discharge were identified. These wetlands provide important habitat diversity and freshwater resources to migratory waterbirds using saline environments (Haig, Murphy, Matthews, Arismendi, & Safeeq, 2019) and were evaluated separately to isolate their surface water trends. Surface water estimates in Bustillos and Babicora monsoonal watersheds occurred from 1990 to 2018 due to a lack of available satellite imagery. Accuracy of surface water area determinations was estimated to be 93%–98% by comparison to the ~20% of lakes/wetlands that overlapped previous work and identical methods used by Donnelly et al. (2019). Accuracy was comparable to similar time‐series wetland inundation studies using Landsat data (Jin et al., 2017).

### 2.3 Lake and wetland change

Changes to lake hydrology were assessed by summarizing SMA results along gradients of averaged variance in annual surface water area and the averaged proportion of basins covered with surface water between two periods 1984–1999 (P1) and 2000–2018 (P2; Figures S1 and S2; Table 2; Tables S1 and S2). We used these two periods primarily to capture the inter‐annual variability of the climate driven by ENSO and PDO, the two main climate teleconnections that control climate in western North America. Two periods also gave a reasonable number of records to produce a statistically valuable results (*n* > 15 years). Maximum surface water area measured from 1984 to 2018 was used to calculate the proportion of lake coverage. Differences were plotted as change vectors for individual lakes grouped by snowmelt and monsoon regions. Wetland change was calculated as the difference in mean area between P1 and P2. Results were partitioned by snowmelt and monsoon watersheds.

**Table 2:** Summary of endorheic watershed change measured as differenced means between 1984–1999 and 2000–2018. Factors included are surface water areas for lakes, wetlands, and peripheral wetlands; area of irrigated agriculture; and human population. Summaries are partitioned by snowmelt (*n* = 18) and monsoonal watersheds (*n* = 8). Far right columns identify number of watersheds with significant change (*p* < .05) by Wilcoxon test and linear regression. All area values are in hectares

	Watersheds	1984–1999	*SD*	2000–2018	*SD*	Change	% Dif	Num. Wilcox‐*p* Sig.	Num. LM‐*p* Sig.
Lakes	Snow	700,109	142,423	509,184	103,450	−190,926	−27	16/18	16/18
	Mons.	41,804	30,168	36,543	25,972	−5,260	−13	1/8	1/8
Wetlands	Snow	141,337	70,010	74,778	44,505	−66,558	−47	16/18	17/18
	Mons.	3,216	2,732	3,448	1,830	231	7	2/8	1/8
Periph. Wetlands	Snow	97,603.9	37,705.7	51,854.8	17,339.7	−45,749.1	−53	ND	ND
	Mons.	NP		NP					
IrrigatedAg.	Snow	878,753	92,796	911,033	82,959	32,280	4	1/18	6/18
	Mons.	338,503	81,419	407,954	81,208	69,452	21	3/8	5/8
Human Pop.	Snow	3,007,420	305,829	4,223,903	408,571	1,216,483	40	18/18	18/18
	Mons.	209,832	28,937	314,669	31,834	104,837	50	8/8	8/8
Totals									
Sum Lakes		741,913		545,727		−196,186	−26	14/26	17/26
Sum Wetlands		327,790.2		213,202.2		−114,588.1	−35	18/26	18/26
Sum Irr. Ag		1,217,256		1,318,988		101,732	8	4/26	11/26
Sum Hum. Pop.		3,217,252		4,538,572		1,321,320	41	26/26	26/26

Abbreviations: ND, not determined; NP, not present.

### 2.4 Estimating factors of human water use

Annual extent of irrigated agriculture and population density change (1984–2018) were utilized as an analog of human water use. Agriculture in arid and semi‐arid regions of western North America accounts for >89% of surface water consumption (Maupin et al., 2010; Vélez & Saez, 2011) as high evaporative demand mandates irrigation for crop production. We assumed all irrigation to impacted ecosystem water balance, but acknowledge different practices (i.e. sprinkler irrigation vs. flood irrigation) and water sources (i.e. surface water vs. groundwater) can influence its effect. Irrigated extent was summarized within endorheic watersheds using Landsat 5 Thematic Mapper (1984–2011) and Landsat 8 Operational Land Imager (2013–2018) satellite imagery. A gap in satellite coverage prevented agricultural monitoring in 2012. Measurements were based on normalized difference vegetation indices (NDVI) as a quantitative measure of high primary productivity (Pettorelli et al., 2005) associated with irrigation. To delineate irrigated area, we generated raster images (30 × 30 m pixel) of maximum NDVI values from overlapping Landsat images. Individual images were representative of the highest primary productivity for each year between 1 February and 30 November. NDVI values >0.4 were considered irrigated (Meier, Zabel, & Mauser, 2018). Summaries were constrained to agricultural fields present in 2018 to remove non‐agricultural NDVI values (e.g. riparian forest). Field boundaries were generated in a GIS by digitizing high‐resolution satellite imagery. The resulting annual time series of irrigated agricultural was normalized by watershed area for purposes of analysis.

Changes in population density were derived from Global Human Settlement Layers, 250 m population grid (Melchiorri & Siragusa, 2018). Population estimates were summarized within endorheic watersheds for available years 1975, 1990, 2000, and 2015. A spline was then fit to the data to estimate remaining years and population density calculated by dividing annual watershed populations (1975–2018) by their watershed area.

### 2.5 Endorheic water balance and climate variables

Under equilibrium conditions, with no external modifications (e.g. no water withdrawal or inter‐basin transfer of water), climate factors control the surface area of lakes through the balance between runoff, precipitation, and evaporation (Budyko, 1974; Mason, Guzkowska, Rapley, & Street‐Perrott, 1994; Mifflin & Wheat, 1979). Because lake area represents a natural equilibrium state between watershed runoff, precipitation, and evapotranspiration, it is also a measure of climate aridity where smaller lake area (for a given watershed) represents more arid conditions and larger lake area represents less arid conditions (Mason et al., 1994). These relationships are unique to endorheic watersheds because their lakes act as a combined ‘rain gauge/evaporation pan’ for the watershed which can be used to determine hydrologic changes that are driven solely by climate (i.e. changes in aridity). Therefore, when observed lake area is lower than that predicted by climate alone, it suggests that human modification of the water budget through basin withdrawals for agriculture and domestic use are present. This distinction makes it possible to attribute human versus climate driven hydrologic change within an endorheic watershed.

Using the SMA results, we explored patterns of lake and wetland surface water area as response variables to hydrologic processes within endorheic watersheds. Area of irrigated agriculture and population density were considered human‐induced predictor variables because they modify the natural water balance through runoff withdrawal and interception. To estimate the attribution of climate versus direct human actions to changes in lake and wetland surface water area, we chose the predictor climate variables that directly affect endorheic water balance: runoff (RO), evapotranspiration (ET), and precipitation (PR); we additionally included snow water equivalent (SWE) as an important component of RO in the snowmelt region of the study (Dierauer, Whitfield, & Allen, 2018; Fritze, Stewart, & Pebesma, 2011).

Endorheic watershed boundaries were defined with the HydroBASINS polygon dataset (Lehner & Grill, 2013) and used to generate annual climate variables extracted from gridded (4 km) monthly TerraClimate data (Abatzoglou, Dobrowski, Parks, & Hegewisch, 2018). TerraClimate was used because of its high spatiotemporal resolution and global coverage derived from interpolation of existing climate data, thus producing a continuous climate records spanning watersheds in the United States and Mexico. Variables were summarized for each watershed within a 12‐month water year; 1 October to 30 September for snowmelt watersheds and 1 April to 31 March for monsoonal watersheds. This regionally staggered approach made it possible to summarize climate factors within annual time periods that were relevant to lake and wetland surface water change. For example, beginning water year 1 October made it possible to capture the effects of winter snowpack on surface water patterns in snowmelt watersheds during spring. For statistical analysis, climate time‐series summaries were generated from 1984 to 2018 to align with SMA surface water estimates. Because TerraClimate data did not cover the full 2018 period for monsoonal watersheds, statistical analyses for this region were conducted only for years 1984–2017. The Summer Lake watershed encompassed watersheds and climate data beyond its endorheic boundary to include areas important to cross‐basin groundwater flow supporting spring discharge that influenced lake area (McFarland & Ryals, 1991).

### 2.6 Statistical analysis

We attributed importance of climate (ET, PR, RO, and SWE) and human water use (irrigated agriculture and population density) to the prediction of lake and wetland surface water area using randomForestSRC regression tree analysis (Ishwaran & Kogalur, 2019), as a nonparametric measure of variable importance (VIMP). This approach is applicable to ecological systems with typically non‐normal distributions, which most of our variables showed through time (Cutler et al., 2007; De'ath & Fabricius, 2000; Zanella, Folkard, Blackburn, & Carvalho, 2017). RandomForestSRC allowed for a two‐step method of randomization to de‐correlate trees, which decreased variance and bias for a stronger representative model (Zhang & Lu, 2012). Confidence intervals for importance measures were calculated using double bootstrap subsampling (*n* = 500, alpha = 0.05; Ishwaran & Lu, 2019) to provide a quantitative view of relative importance for each variable in the model. The Breiman–Cutler (aka, permutation) method of VIMP calculations was applied to all random forest analysis (Breiman, 2002). Model runs were conducted using 5,000 trees. Variable rankings were presented as boxplots for lakes and wetlands, partitioned by snowmelt and monsoonal regions.

We quantified change to climate variables (ET, PR, RO, and SWE) and lake surface area using the nonparametric Wilcoxon test (Siegel, 1957). Data were binned temporally from 1984 to 1999 (P1) and 2000 and 2018 (P2) to compare differences in long‐term trends. *p* value is considered a measure of significance strength in the difference between the two periods, but for convenience we used a *p* value of .05 to represent significant/nonsignificant change. Boxplots were used to visualize variability and change and are provided in the Supporting Information (Figures S1–S10).

### 2.7 Data processing

All image processing and raster‐based analyses were conducted using Google Earth Engine cloud‐based geospatial processing platform (Gorelick et al., 2017). Landsat images used were calibrated across sensors and corrected for atmospheric effects and illumination/viewing geometry (Masek et al., 2006; Vermote, Justice, Claverie, & Franch, 2016). All GIS analysis was completed using QGIS (QGIS Development Team, 2017). Plotting and statistical analyses were completed using the R environment (RStudio Team, 2016; R Core Team, 2018), including the R‐packages, randomForestSRC (Ishwaran & Kogalur, 2019), and Tidyverse (Wickham, 2017).

## 3 RESULTS

Detailed data supporting our analyses of waterbird flyway resilience in western North American are provided in Tables S1–S10 and Figures S1–S16, for all variables presented below. Annual (1984–2018) surface water change and human‐climate factors were aggregated into two time periods 1984–1999 (P1) and 2000–2018 (P2) for watershed level comparisons. All change values presented are differences between P1 and P2 means with significant *p* values ≤.05 derived from Wilcoxon ranked order test unless otherwise noted.

### 3.1 Surface water trends in endorheic watersheds

**Figure 2:**
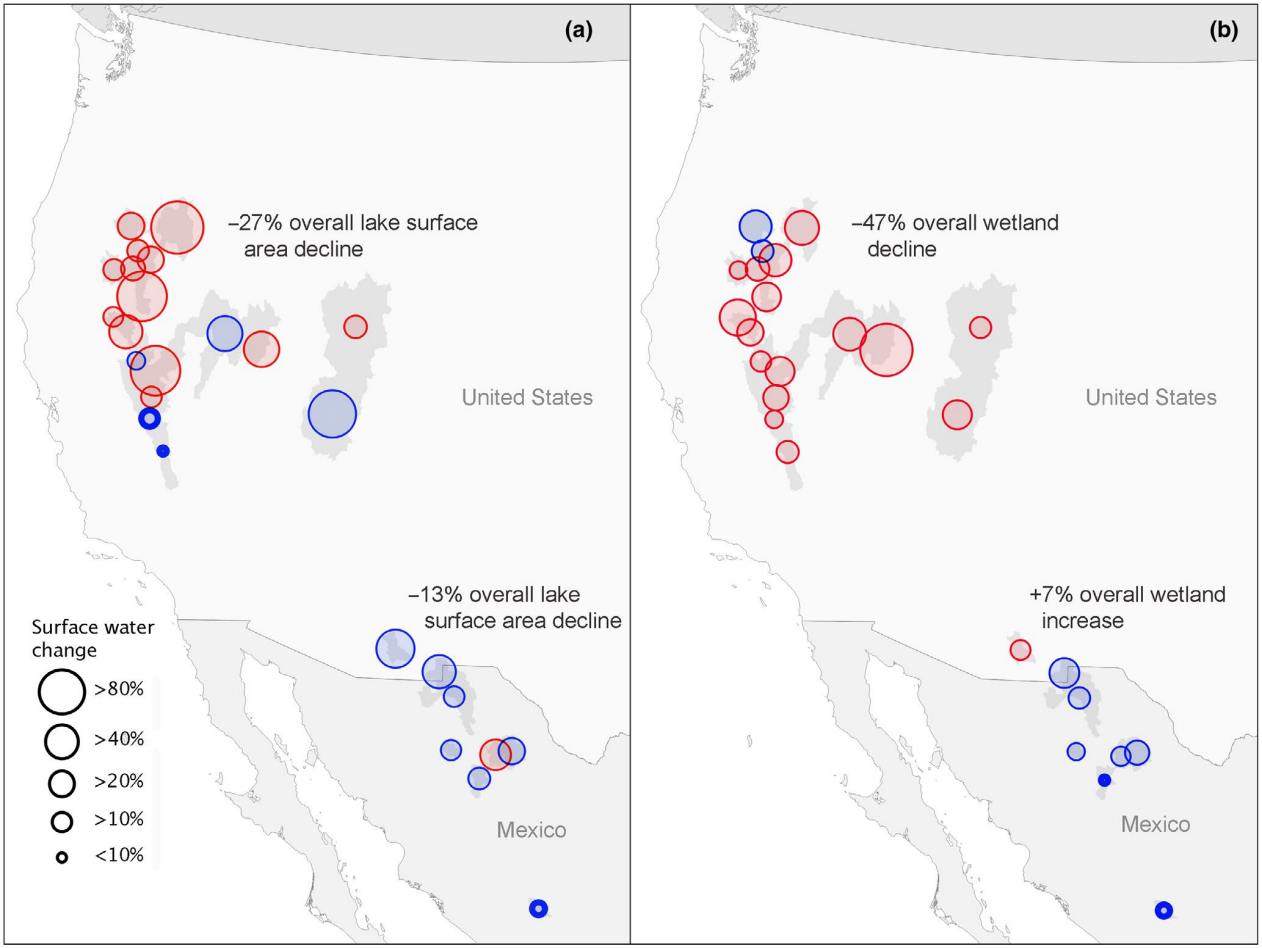
Magnitude of surface water change for 26 endorheic watersheds between 1984–1999 and 2000–2018. Change is partitioned by lakes (a) and wetlands (b). Statistically significant (*p* < .05) declines are shown in red and insignificant declines shown in blue. Increases to surface water area are shown in bold blue outline

Long‐term monitoring of surface water revealed that lakes in snowmelt watersheds diminished at twice the rate (−27%, ~191 kha) as those found in monsoonal watersheds (−13%, ~5.3 kha; Figure 2a; Table 2). Whereas all lakes showed substantial interannual variability, patterns in long‐term trends differed substantially between snowmelt and monsoonal regions. Lake area declines were significant in 13 of 18 snowmelt watersheds (Figure 2a; Figure S1; Table S1) with most annual trends exhibiting strong linear declines from 1984 to 2018 (Figure S9). Additionally, nearly significant lake area declines (*p* < .08) were recorded in Humboldt and Sevier snowmelt watersheds. In contrast, lake area change was significant in only one of eight monsoonal watersheds with linear trends generally showing insignificant declines (Figure 2a; Figures S1 and S9; Table S1). Outliers included Mono and Owens Lakes in snowmelt watersheds and Laguna de Santiaguillo in monsoonal watersheds, all of which gained surface water area over time (Table S1).

**Figure 3:**
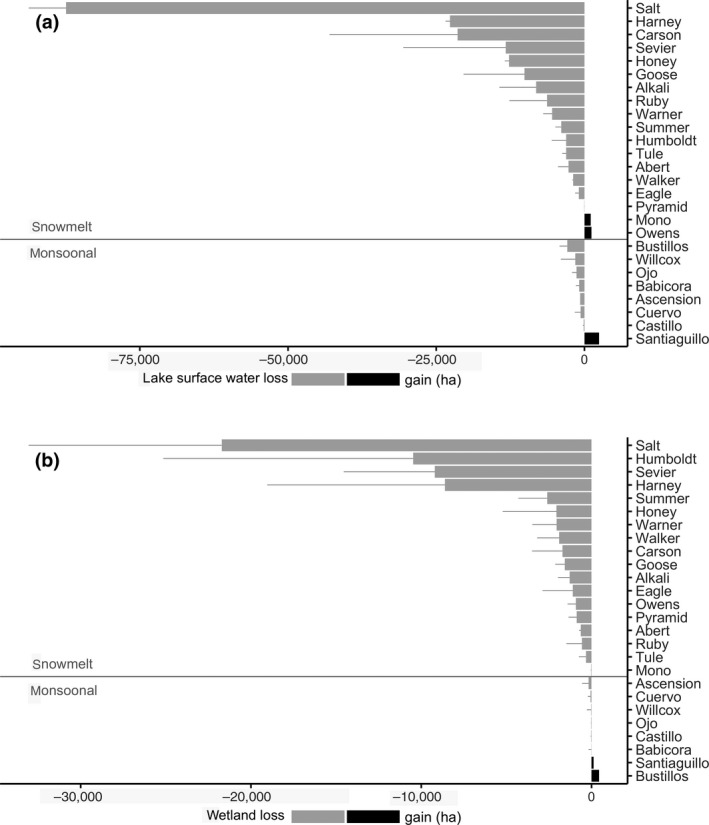
Change in mean endorheic lake (a) and wetland (b) surface water area between periods, 1984–1999 and 2000–2018. Results partitioned by snowmelt and monsoonal watersheds

Designated hemispherically and globally important waterbird sites in snowmelt watersheds, Lake Abert, Carson Sink, Harney Basin, and Great Salt Lake (Kushlan et al., 2002; Senner et al., 2016), diminished by −17% (−2.6 kha), −90% (−21.3 kha), −59% (−14.2 kha), and −21% (−85.1 kha), respectively (Figure 3; Table S1). Similarly, monsoonal Ramsar designations of international importance Laguna de Babicora declined by −11% while Laguna de Santiaguillo increased by +24% (Figure 3; Table S1; Pérez‐Arteaga, Gaston, & Kershaw, 2002).

**Figure 4:**
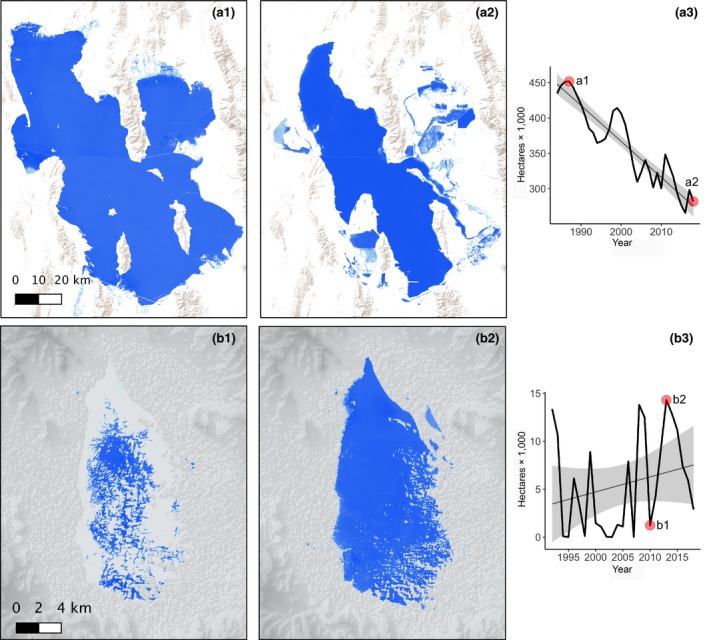
Annual time series surface water model example for Great Salt Lake, Utah, USA (a) and Laguna de Babicora, Chihuahua, Mexico (b). Graphs (a3, b3) depict patterns characteristic of lake trends in snowmelt (a) and monsoonal (b) watersheds. The thin black lines are the least squares regression lines with the 0.95% confidence interval of the slope shown in gray fill. Red points identify the first and last image of the period of lake conditions depicted (a1 = 1985, a2 = 2018; b1 = 2010, b2 = 2014)

Overall annual variability in lake surface water area differed between snowmelt and monsoonal watersheds as characterized regionally by patterns observed at Great Salt Lake and Laguna de Babicora (Figure 4). Surface water area at Great Salt Lake, for example, showed a strong linear decline over time, where long‐term change (1984–2018) outweighed near‐term variability (Figure 4a). In contrast, annual surface water variability at Laguna de Babicora was much greater than the long‐term change (Figure 4b), meaning that extensive surface water coverage or near dryness was equally likely from year to year so that long‐term change was outweighed by near term variability. While monsoonal patterns were found to be highly dynamic, over the long term they remained relatively stable. Snowmelt patterns generally showed long‐term declines with relatively small short‐term change.

Changing patterns of wetland coverage were comparable to those observed in lakes. Wetlands within snowmelt watersheds lost −47% (~67 kha) of surface water area, while wetted area in monsoonal watersheds increased slightly (+7%, ~200 ha) as declines in some watersheds were offset by gains in others (Figures 2b and 3; Table 2). Wetland drying was significant in 16 of 18 snowmelt watersheds (Figure 2b; Table S2) with substantial declines to watersheds supporting four globally and hemispherically important waterbird sites: Abert, −20% (−628 ha); Carson, −43% (−1,712 ha); Harney −56% (−8,605 ha); and Great Salt Lake, −18% (−20,629 ha; Figure 3; Table S2). Total wetland area differed substantially by region as monsoonal watersheds on average contained only ~3% (~3,000 ha) of the wetland area found in snowmelt watersheds (~100 kha). High variability and small wetland footprint made most change in monsoonal watersheds statistically insignificant. Only Bustillos showed a significant increase (+51%) and Willcox Playa a significant decline (−12%; Table S2).

Over half (61%, 65 kha) of flooded wetlands in snowmelt watersheds were coincident with irrigated agriculture from 1984 to 2018, but occurred on only 7% of irrigated lands. In a post‐hoc assessment, we determined these agriculturally related wetlands to be associated with flood irrigation of riparian wet meadows used for livestock forage (Peck, McLeod, Hewlett, & Lovvorn, 2004). Non‐agricultural snowmelt wetlands (39%, 41.2 kha) occurred naturally or were sites flooded specifically for wildlife (e.g. public wildlife refuges; Table S10). In monsoonal watersheds, earthen dams constructed across ephemeral drainages to capture storm runoff for livestock watering made up over two‐thirds (67%, 2.0 kha) of flooded wetland areas (Table S10). Our inventory of wetland features identified 1,908 small agricultural dams in monsoonal watersheds. Dams occurred in upper watersheds as opposed to irrigated agricultural and terminal lakes that occupied valley bottoms.

We found peripheral freshwater wetlands (those fed mostly from groundwater, adjacent to larger lakes/wetland systems) in 7 of 26 endorheic lakes, all in snowmelt watersheds (Table S3). The area of these systems declined −53% from ~98 to ~52 kha (Table 2; Table S3). Loss was substantial in designated global and hemispherically important waterbird sites: Carson Sink (−41%, −2.9 kha), Harney Basin (−81%, −6.8 kha), and Great Salt Lake (−44%, −34.7 kha). Summer Lake was the only site where peripheral wetland area increased, but only by a small amount (+2%, 19 ha).

### 3.2 Human water use and climate trends

Irrigated agriculture increased from ~879 to ~911 kha (+4%) and from ~339 to ~408 kha (+21%) in snowmelt and monsoonal watersheds (Table 2). Increases were significant in one of 18 snowmelt watersheds (Summer Lake, +13%) and three of eight monsoonal watersheds (Ascensión, +108%, +40.2 kha; Castillo, +709%, +5.7 kha; and Willcox Playa +43%, +6.4 kha; Table S4).

Human population density, a surrogate used for domestic/industrial water consumption, increased by +41% (~1.3 million people) across all watersheds and by +40% (~1.2 million people) and +50% (~105 thousand people) in snowmelt and monsoonal watersheds individually (Table 2). Growth was significant (*p* < .001) in all but the snowmelt‐watershed Harney Basin, where populations declined significantly (Table S5). Despite growth, human population densities remained relatively low in most areas; however, changes were likely indicative of increasing domestic/industrial water demand throughout the study area.

Of climate variables examined as potential lake and wetland surface water predictors, ET was the only variable to increase significantly (+6%) across all snowmelt watersheds (Table 3; Table S6). Linear time‐series trends for ET were also statistically significant for the same watersheds (Figure S13; Table S6). In contrast, changes to ET were insignificant in all monsoonal watersheds, increasing by only +1% overall (Table 3; Table S6). Increasing linear trends, however, were significant in seven of eight monsoonal watersheds (Figure S13).

**Table 3:** Summary of changing evapotranspiration (ET), precipitation (PR), runoff (RO), and snow water equivalent (SWE) means within snowmelt and monsoonal watersheds between 1984–1999 and 2000**–**2018. Far right columns identify number of basins with significant change (*p* < .05) by Wilcoxon test and linear regression. All units are in mm. See Tables S6–S9, Figures S5–S8 and S13–S16 for details

	Water sheds	1984–1999	*SD*	2000–2018	*SD*	Change	% Dif	Num. Wilcox‐*p* Sig.	Num. LM‐*p* Sig.
ET	Snowmelt	1,108	60	1,172	47	64	6	18/18	18/18
	Monsoonal	1,616	52	1,635	44	19	1	0/8	7/8
PR	Snowmelt	376	107	327	88	−50	−13	3/18	3/18
	Monsoonal	420	98	416	90	−4	−1	0/8	1/8
RO	Snowmelt	104	66	80	58	−24	−23	0/18	0/18
	Monsoonal	25	13	27	16	2	8	0/8	0/8
SWE	Snowmelt	310	151	264	129	−46	−15	0/18	0/18
	Monsoonal	0.16	0.15	0.08	0.06	0	−54	0/8	1/8

Changes to climate variables, PR, RO, and SWE, were largely insignificant (Figures S6–S8 and S13–S16; Table 3; Tables S6–S9). Precipitation (PR) change was significant in only 3 of 18 snowmelt watersheds, declining −13% overall (Table 3). Precipitation remained statistically unchanged in all monsoonal watersheds, declining −1% (Table 3). Changes to RO were statistically insignificant in all watersheds, but changed by −23% and +8% in snowmelt and monsoonal watersheds, respectively (Table 3). SWE remained statistically unchanged in all snowmelt watersheds, but declined −15% (Table 3). Only monsoonal watershed Willcox Playa had measurable SWE. Annual accumulation was minimal and showed no significant change. See Supporting Information figures and tables cited above for detailed Wilcoxon comparisons or linear trends (1984–2019) for all climate variables.

### 3.3 Predictor variable importance

**Figure 5:**
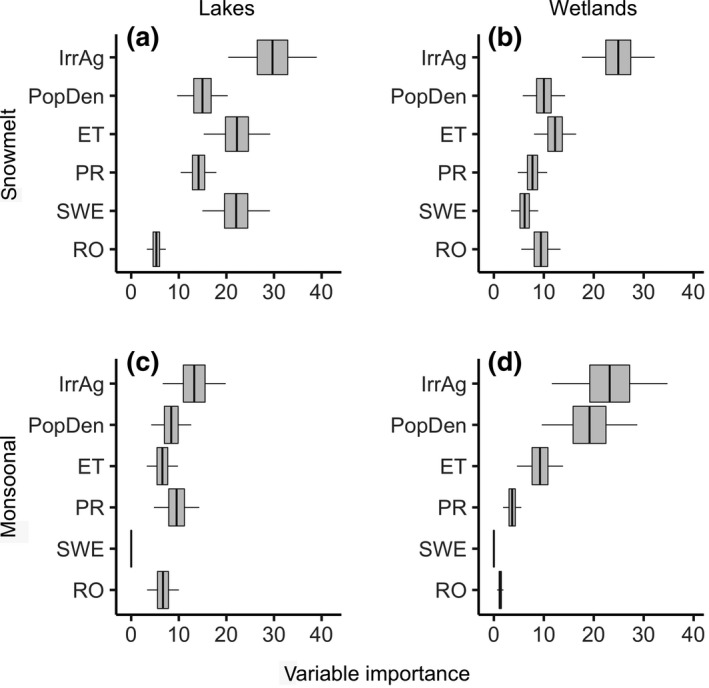
Predictive variable importance (VIMP) derived from randomForest regression analysis of annual surface water area (1984–2018) for endorheic lakes and wetlands within snowmelt and monsoonal watersheds (see Figure 1). Box: 25th, 50th (heavy vertical line), and 75th percentiles. Whiskers: 5th and 95th percentiles. Predictor variables: ET, evapotranspiration; IrrAg, irrigated agricultural area; PopDen, human population density; PR, precipitation; RO, runoff; SWE, snow water equivalent. VIMP is derived from Breiman‐Cutler permutation; see Section 2 for details); VIMP is standardized for comparisons among all plots

Variable importance analyses identified irrigated agriculture as the most important predictor of lake and wetland surface water, although its level of importance varied (Figure 5). Irrigated agriculture was the dominant driver of wetland area with VIMP scores double that of climate variables (Figure 5b,d). Population density was also an important predictor of wetland surface water in monsoonal watersheds (Figure 5d). While irrigated agriculture was the single most important predictor of lake surface area, individual climate variables were nearly as important (Figure 5a,c). SWE was inconsequential to monsoonal lakes and wetlands do to its rarity in the region (Figure 5c,d).

## 4 DISCUSSION

This study is the first that we are aware of to assess long‐term lake and wetlands trends in endorheic watersheds spanning the United States and Mexico. Outcomes document strengthening patterns of landscape desiccation that could have major effects on waterbird habitats implicating a greater need for focused wetland conservation in this expansive region. Lake and wetland surface water loss was pervasive, driven largely by agricultural water use coupled with rising evaporative demands. Trends were particularly concerning in snowmelt watersheds that supported the vast majority of wetland resources where drying was significant in 16 of 18 watersheds. While monsoonal watersheds represented a smaller proportion of total lake and wetland area, their distribution within highly arid regions of the continental flyway increases their value to migratory waterbirds. Less predictable annual patterns of inundation among these watersheds due to spatial variance in monsoonal rainfall were offset by broader habitat stability. However, long‐term wetland inundation in the region remained relatively constant and generally highly productive when wet.

Wetland relationships with farming and ranching practices are complex and the simple interpretation that all irrigated agriculture is detrimental for wetlands is over generalized and likely misleading. In monsoonal watersheds, small earthen dams meant to capture surface runoff for livestock watering were a major component of wetland resources (67%). When flooded, dams acted as an artificial network of isolated wetlands known to support 31 species of waterbirds (Riojas‐López & Mellink, 2005). We speculate through our results that increased wetland inundation within monsoonal watersheds was in‐part due to new dam construction associated with growing human populations (+50%) and livestock ranching in the region. Rapid expansion of irrigated agriculture identified in monsoonal watersheds (+21%) was attributed to new and unregulated groundwater use in valley bottoms (Pool, Panjabi, Macias‐Duarte, & Solhjem, 2014). Despite increasing hydrologic perturbation (e.g. damming and groundwater extraction) in many watersheds, there were no clear pattern modifications for decreasing areas of surface water. Area of lake and wetland coverage (natural and dammed) remained variable suggesting a stronger climatic influence.

A total of 61% of wetlands within snowmelt watersheds were affiliated with irrigated agriculture, mainly consisting of flooded riparian wet meadows which are a valuable waterbird habitat (Fleskes & Gregory, 2010; McWethy & Austin, 2009; Moulton, Carlisle, Brenner, & Cavallaro, 2013). Despite these contributions, only 7% of irrigated lands within snowmelt watersheds was associated with wetlands. Clearly, how and where irrigation is implemented has important consequences to wetland availability. We lacked local irrigation and crop level data in watersheds to fully understand the mechanisms behind patterns of wetland drying that may be associated with agricultural practices. However, previous studies suggest changes to crop type (Bishop, Curtis, & Kim, 2010) and irrigation regimes (Hassanli, Ebrahimizadeh, & Beecham, 2009; Pfeiffer & Lin, 2014) can affect overall water use. Ironically, well‐intended water savings practices, such as drip and sprinkler irrigation, have been linked to increased water consumption, diminished stream return flows, and lower aquifer recharge rates (Scott, Vicuña, Blanco, Meza, & Varela, 2014; Ward & Pulido‐Velazquez, 2008).

**Figure 6:**
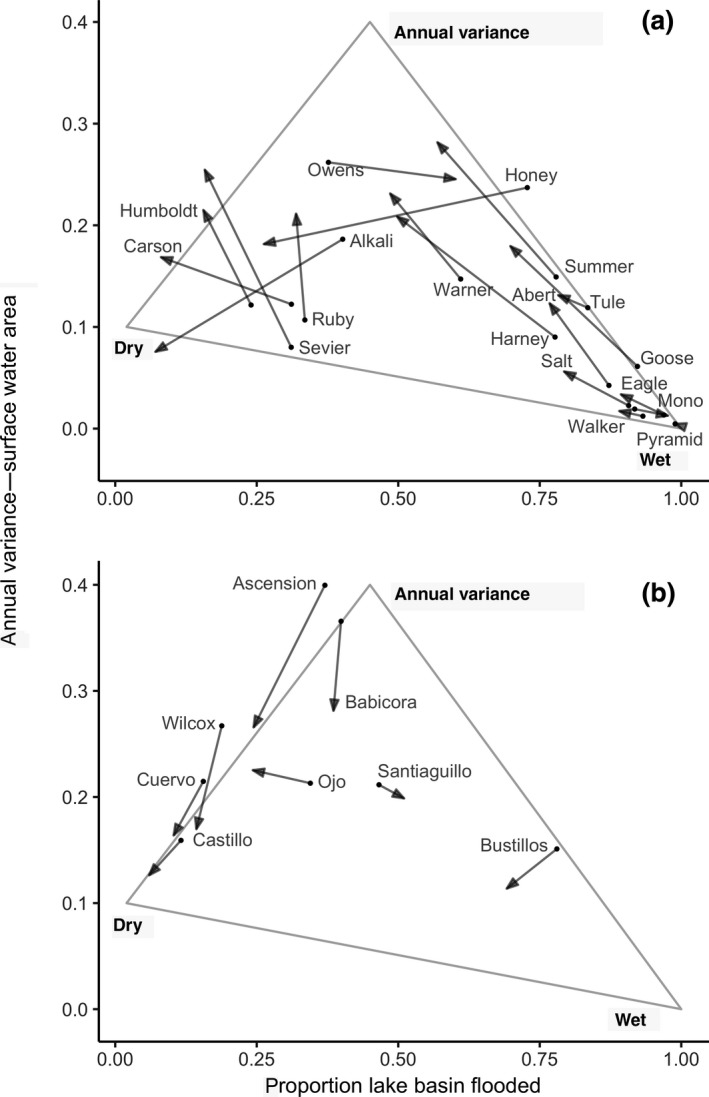
Transition in lake hydrology along gradients of averaged variance in annual surface water area and averaged proportion of lake basins inundated between two periods, 1984–1999 and 2000–2018. Data are partitioned by snowmelt (a) and monsoonal (b) watersheds. Change is measured as a vector depicting the rate and direction of transition between periods along a continuum of states from ‘wet’, ‘dry’, and ‘annual surface water variance’. Maximum surface water area measured from 1984 to 2018 was used to calculate the proportion of lake inundated

Sustained patterns of lake declines across snowmelt watersheds (Figure 6) suggest a regional tipping‐point in ecosystem water balance has been reached where increasing human and evaporative demands now consistently overdraw water supplies. Declining surface water trends raises concerns of trophic collapse within watersheds supporting productive saline lake food webs (e.g. Lake Abert, Mono Lake, and Great Salt Lake). Lower lake levels are resulting in increased salinity rates as freshwater inflows diminish (Larson et al., 2016; Moore, 2016). Higher salinity can drastically reduce diversity and biomass of benthic macroinvertebrates that serve as critical food resource for waterbirds. As water volumes continue to decrease, lakes can reach a point of infertility well before they dry completely (Herbst, 2006; Senner et al., 2018). Transition of some declining freshwater lakes to saline states (sensu Thomas, 1995—Walker Lake) may open habitat niches in snowmelt watersheds that offset losses in others. However, these lakes may also be vulnerable to collapse if freshwater inflows continue to decline.

### 4.1 Implications to waterbird conservation

The results of this study have significant implications to waterbirds at both local and landscape levels because of (a) the overall impacts of water use and climate on lake and wetland area, (b) increasing lake salinity, and (c) broad scale loss of migratory connectivity along waterbird flyways. The significant decline in wetland area across snowmelt watersheds (−47%) is disconcerting. Comparatively, lake area in monsoonal watersheds were more stable with small overall increases in wetland extent. However, declining water quality has been identified as a conservation challenge in some monsoonal watersheds that may further deteriorate flyway resilience due to increased pollution from urbanization and industrialized agriculture (Benavides et al., 2008). Additionally, expanding agriculture in monsoonal watersheds is being driven by groundwater pumping (Pool et al., 2014) and long‐term consequences of this practice on wetland resilience remains unclear. Some lakes (McFarland & Ryals, 1991) and wetlands (Downard & Endter‐Wada, 2013) rely on groundwater as a significant component of their water budget and groundwater declines could affect their long‐term condition and functionality as waterbird habitat (Pritchett & Manning, 2012).

While groundwater connections are unknown, it was clear that monsoon rainfall patterns were an important driver of wetland area in monsoonal watersheds. Analysis of future climate in the Mexican Highlands predicts precipitation from 2030 to 2045 to be variable, but remains similar to patterns of recent decades (Verduzco et al., 2018). Although Cook and Seager (2013) similarly found that by 2080–2099 total precipitation would remain approximately the same, the timing of peak monsoon rainfall would shift from June–July to September–October. In addition, they found that despite stable precipitation trends, rising temperature and evapotranspiration would lead to increased annual drying. These changing monsoonal patterns coupled with increased evapotranspiration will likely shape lake and wetland resiliency in monsoonal watersheds by altering the timing and volume of runoff, influencing waterbird habitat availability and agricultural and urban water use.

In addition to loss and altered timing of runoff to wetlands in general, the specific loss of freshwater wetlands along the periphery and adjacent to lakes raises concern over potential decline of ecosystem diversity within some snowmelt watersheds (Table S10). In saline lakes particularly, waterbirds are reliant on adjacent freshwater wetlands to balance physiological demands of saltwater environments (i.e. osmoregulation), especially during brood rearing when chicks are freshwater dependent (Rocha et al., 2016; Wollheim & Lovvorn, 1995). Loss of peripheral wetlands was greater (−53%) than those in upper watersheds (−47%) and likely a result of compounding upstream water diversions. In agriculturally dominated snowmelt watersheds, drought effects are not evenly distributed as water is allocated hierarchically on a ‘first in time, first in right’ basis where long‐time irrigators are granted priority rights to consume water apportionments prior to junior users (Getches, Zellmer, & Amos, 2009). Depending on individual state laws, however, pumping of groundwater may or may not be regulated and can affect surface flows because of groundwater and surface water connectivity (Cooper, Sanderson, Stannard, & Groeneveld, 2006). Furthermore, legal rights to protect in‐stream flows and associated ecosystem services were unrecognized as a beneficial use until the 1970s (Benson, Dan, Corbridge, Getches, & Bates, 2014) and are legally cumbersome in some states today (Szeptycki, Forgie, Hook, Lorick, & Womble, 2015), resulting in low priority and diminished water availability for maintenance of natural wetland systems.

Hydrologic resilience of western North American flyways has allowed pioneering waterbirds to leverage wetland availability to offset drought and maintain connectivity by adapting migratory pathways to shifting continental resource conditions (Albanese & Davis, 2013; Skagen, Granfors, & Melcher, 2008). Lake and wetland declines we identify in snowmelt watersheds may signal a loss of plasticity in migratory networks. Further impacts are expected as forecast of drought and water use demands intensify over coming decades (Dettinger, Udall, & Georgakakos, 2015). Drying of individual lakes has the potential of dramatically reconfiguring energetic demand of migration by increasing the flight distances between stopovers and reducing the total number of sites available to birds in water scarce landscapes (Haig et al., 1998). Some waterbirds have demonstrated adaptive capacity to mitigate changing resource distributions during breeding and migration (Rakhimberdiev et al., 2018), but it is unclear if these traits can accommodate projected landscape conditions.

In contrast, relatively stable lake and wetland extent in monsoonal wintering grounds and southern migration routes indicate a nonlinear response to shifting climatic norms along flyway latitudes (sensu Seddon, Macias‐Fauria, Long, Benz, & Willis, 2016). Changing ecological trends underscore the importance of assessing range‐wide impacts to migratory bird habitats (Kirby et al., 2008), which in the past have focused primarily on northern breeding grounds (Small‐Lorenz et al., 2013). While breeding success is identified as an important vital rate (Douglas & Pearce‐Higgins, 2014; Koons, Gunnarsson, Schmutz, & Rotella, 2014), wetland drying in key waterbird migration sites could trigger new and powerful ecological bottlenecks that limit populations (Murray et al., 2018; Xu et al., 2019). The spatial location of suitable wetland resources and their temporal availability relative to the migration ecology of individual species are necessary to assess migratory connectivity. A lack of suitable resources, whether through wetland loss, altered timing of wetland availability, unsuitable salinity, or water depths could result in reduced fitness and continental migration failure. In this study, we assessed only wetland and lake availability and not habitat quality. Thus, lake and wetland area estimates could overstate potential waterbird habitat resources and finer scale habitat quality assessments are needed to fully understand habitat connectivity for a given species. However, this study provides a strong foundation to begin assessment of flyway resiliency for a variety of waterbird species.

### 4.2 Finding solutions

Sustainability of waterbird migration flyways in western North America will require adaptive changes to existing conservation priorities (e.g. North American Wetlands Conservation Act *North American Wetlands Conservation Act*, 1989) considerate of accelerating lake and wetland drying. To date, waterbird conservation has been structured around policies to protect land designated as wetlands (e.g. McBeth, 1997; Farm Bill swamp buster provisions) rather than the water supplies crucial to wetland hydrologic function (Downard & Endter‐Wada, 2013). Evolutions in urban planning are offering solutions demonstrated by water efficiency programs and flexibility in water supply development used by the city of Los Angeles, California, for example, to reduce their reliance on Mono and Owens Lakes diversions by 60% (Hughes, Pincetl, & Boone, 2013). Forward looking voluntary and incentive‐based approaches to agricultural water use could have similar effects, wherein producers are supported through government cost sharing of more efficient irrigation infrastructure and, in turn, are compensated for water savings designated for maintenance of wetland habitats (Castle, Beattie, Smith, Peternell, & Kowalski, 2016; Grafton et al., 2018). It is critical, however, that new water savings be redirected to ecosystem services (Kendy et al., 2018) as numerous studies indicate that irrigation efficiency often leads to planting of more water‐intensive crops or expansion of agricultural areas (Batchelor et al., 2014; Scott et al., 2014).

We make our data available to landscape planners to promote ecosystem water balance of agriculture, urban, and waterbird migration. Applications may include targeted preservation of irrigation practices supporting wetlands that made up only 7% of the agricultural footprint in snowmelt watersheds, but were associated with the majority of freshwater emergent wetlands. Flood irrigation of these sites are often perceived as wasteful and singled out by water efficiency efforts as a means to generate agricultural water savings used to offset growing urban demands (Richter et al., 2017). Such practices, however, can unintentionally accelerate wetland loss and eliminate waterbird habitats that further degrade migratory flyways (Ward & Pulido‐Velazquez, 2008). Consideration of the specific social, ecological, economic and hydrological contexts of watersheds and underlying aquifers will be necessary to accurately identify impacts and opportunities of various water management decisions. As noted in this study, the conservation value of these wetlands to waterbirds is manifested in the context of both local waterbird habitat needs and their contribution to processes supporting broader migratory connectivity. We encourage the use of our results to inform conservation solutions by means of collaborative and proactive decision‐making among local and international stakeholders throughout western North American flyways.

## CONFLICT OF INTEREST

The authors declare that there is no conflict of interest.

## Data Availability

The data that support the findings of this study are available from the corresponding author upon reasonable request.

## References

[gcb15010-bib-0001] Abatzoglou, J. T. , Dobrowski, S. Z. , Parks, S. A. , & Hegewisch, K. C. (2018). TerraClimate, a high‐resolution global dataset of monthly climate and climatic water balance from 1958–2015. Scientific Data, 5(170191), 1–12. 10.1038/sdata.2017.191 29313841 PMC5759372

[gcb15010-bib-0002] Adams, D. K. , & Comrie, A. C. (1997). The North American Monsoon. Bulletin of the American Meteorological Society, 78(10), 2197–2213. 10.1175/1520-0477(1997)078<2197:TNAM>2.0.CO;2

[gcb15010-bib-0003] Adams, J. B. , & Gillespie, A. R. (2006). Remote sensing of landscapes with spectral images: A physical modeling approach. Cambridge, UK: Cambridge University Press, 388 pp.

[gcb15010-bib-0004] Albanese, G. , & Davis, C. A. (2013). Broad‐scale relationships between shorebirds and landscapes in the Southern Great Plains. The Auk, 130(1), 88–97. 10.1525/auk.2012.11240

[gcb15010-bib-0005] Baldassarre, G. A. (2014). Ducks, geese, and swans of North America. Baltimore, MD: JHU Press, 1027 pp.

[gcb15010-bib-0006] Batchelor, C. , Reddy, V. R. , Linstead, C. , Dhar, M. , Roy, S. , & May, R. (2014). Do water‐saving technologies improve environmental flows? Journal of Hydrology, 518, 140–149. 10.1016/j.jhydrol.2013.11.063

[gcb15010-bib-0007] Benavides, A. , Moreno, M. , Sosa, M. , Puga, S. , Alcalá, J. , & Quintana, C. (2008). Water quality assessment in the main lagoons of the state of Chihuahua. Revista Latinoamericana de Recursos Naturales, 4(2), 84–88. Retrieved from https://sswm.info/sites/default/files/reference_attachments/BENAVIDES%2520et%2520al%25202008.%2520Evaluaci%25C3%25B3n%2520de%2520la%2520calidad%2520del%2520agua%2520en%2520lagunas%2520edo.%2520Chihuahua.pdf

[gcb15010-bib-0008] Benson, R. D. , Dan Tarlock, A. , Corbridge, J. N. , Getches, D. H. , & Bates, S. F. (2014). Water resource management. St. Paul, MN: Foundation Press, 911 pp. Retrieved from https://digitalrepository.unm.edu/law_facbookdisplay/33

[gcb15010-bib-0009] Bishop, C. D. , Curtis, K. R. , & Kim, M.‐K. (2010). Conserving water in arid regions: Exploring the economic feasibility of alternative crops. Agricultural Systems, 103(8), 535–542. 10.1016/j.agsy.2010.05.006

[gcb15010-bib-0010] Boere, G. C. , & Stroud, D. A. (2006). The flyway concept: What it is and what it isn't In Boere G. C., Galbraith C. A., & Stroud D. A. (Eds.), Waterbirds around the world (pp. 40–47). Scotland, UK: The Stationery Office Edinburgh.

[gcb15010-bib-0011] Box, E. O. , Holben, B. N. , & Kalb, V. (1989). Accuracy of the AVHRR vegetation index as a predictor of biomass, primary productivity and net CO_2_ flux. Vegetatio, 80(2), 71–89. 10.1007/BF00048034

[gcb15010-bib-0012] Breiman, L. (2002). Manual on setting up, using, and understanding random forests v3.1. Berkeley, CA: Statistics Department University of California, 29 pp. Retrieved from https://www.stat.berkeley.edu/~breiman/Using_random_forests_V3.1.pdf

[gcb15010-bib-0013] Budyko, M. I. (1974). Climate and life. New York, NY: Academic Press, 507 pp.

[gcb15010-bib-0014] Buehler, D. M. , & Piersma, T. (2008). Travelling on a budget: Predictions and ecological evidence for bottlenecks in the annual cycle of long‐distance migrants. Philosophical Transactions of the Royal Society B: Biological Sciences, 363(1490), 247–266. 10.1098/rstb.2007.2138 PMC2606749 17638692

[gcb15010-bib-0015] Castle, A. , Beattie, A. , Smith, Z. , Peternell, D. , & Kowalski, T. (2016). Improving irrigation water uses for agricultural and environmental benefits. Boulder, CO: University of Colorado, 39 pp. Retrieved from https://scholar.law.colorado.edu/cgi/viewcontent.cgi?article=1181%26context=books_reports_studies

[gcb15010-bib-0016] Cook, B. I. , & Seager, R. (2013). The response of the North American Monsoon to increased greenhouse gas forcing. Journal of Geophysical Research: Atmospheres, 118(4), 1690–1699. 10.1002/jgrd.50111

[gcb15010-bib-0017] Cooper, D. J. , Sanderson, J. S. , Stannard, D. I. , & Groeneveld, D. P. (2006). Effects of long‐term water table drawdown on evapotranspiration and vegetation in an arid region phreatophyte community. Journal of Hydrology, 325(1), 21–34. 10.1016/j.jhydrol.2005.09.035

[gcb15010-bib-0018] Cutler, D. R. , Richard, C. D. , Edwards, T. C. , Beard, K. H. , Cutler, A. , Hess, K. T. , … Lawler, J. J. (2007). Random forests for classification in ecology. Ecology, 88(11), 2783–2792. 10.1890/07-0539.1 18051647

[gcb15010-bib-0019] Dai, A. (2013). Increasing drought under global warming in observations and models. Nature Climate Change, 3(1), 52–58. 10.1038/nclimate1633

[gcb15010-bib-0020] De'ath, G. , & Fabricius, K. E. (2000). Classification and regression trees: A powerful yet simple technique for ecological data analysis. Ecology, 81(11), 3178–3192. 10.1890/0012-9658(2000)081[3178:CARTAP]2.0.CO;2

[gcb15010-bib-0021] Dettinger, M. , Udall, B. , & Georgakakos, A. (2015). Western water and climate change. Ecological Applications, 25(8), 2069–2093. 10.1890/15-0938.1 26910940

[gcb15010-bib-0022] DeVries, B. , Huang, C. , Lang, M. W. , Jones, J. W. , Huang, W. , Creed, I. F. , & Carroll, M. L. (2017). Automated quantification of surface water inundation in wetlands using optical satellite imagery. Remote Sensing, 9(8), 807 10.3390/rs9080807

[gcb15010-bib-0023] Devries, J. H. , Brook, R. W. , Howerter, D. W. , & Anderson, M. G. (2008). Effects of spring body condition and age on reproduction in Mallards (*Anas platyrhynchos*). The Auk, 125(3), 618–628. 10.1525/auk.2008.07055

[gcb15010-bib-0024] Dierauer, J. R. , Whitfield, P. H. , & Allen, D. M. (2018). Climate controls on runoff and low flows in mountain catchments of Western North America. Water Resources Research, 54(10), 7495–7510. 10.1029/2018WR023087

[gcb15010-bib-0025] Donnelly, J. P. , Naugle, D. E. , Collins, D. P. , Dugger, B. D. , Allred, B. W. , Tack, J. D. , & Dreitz, V. J. (2019). Synchronizing conservation to seasonal wetland hydrology and waterbird migration in semi‐arid landscapes. Ecosphere, 10(6), 1–12. 10.1002/ecs2.2758

[gcb15010-bib-0026] Douglas, D. J. T. , & Pearce‐Higgins, J. W. (2014). Relative importance of prey abundance and habitat structure as drivers of shorebird breeding success and abundance. Animal Conservation, 17(6), 535–543. 10.1111/acv.12119

[gcb15010-bib-0027] Downard, R. , & Endter‐Wada, J. (2013). Keeping wetlands wet in the western United States: Adaptations to drought in agriculture‐dominated human‐natural systems. Journal of Environmental Management, 131, 394–406. 10.1016/j.jenvman.2013.10.008 24211568

[gcb15010-bib-0028] Drewien, R. C. , Brown, W. M. , & Benning, D. S. (1996). Distribution and abundance of sandhill cranes in Mexico. The Journal of Wildlife Management, 60(2), 270–285. 10.2307/3802225

[gcb15010-bib-0029] Drewien, R. C. , Terrazas, A. L. , Taylor, J. P. , Barraza, J. M. O. , & Shea, R. E. (2003). Status of lesser snow geese and Ross's geese wintering in the interior highlands of Mexico. Wildlife Society Bulletin, 31(2), 417–432.

[gcb15010-bib-0030] Ellis, H. I. , & Jehl, J. R. Jr. (2003). Temperature regulation and the constraints of climate in the Eared Grebe. Waterbirds, 26(3), 275–279. 10.1675/1524-4695(2003)026[0275:TRATCO]2.0.CO;2

[gcb15010-bib-0031] Fleskes, J. P. , & Gregory, C. J. (2010). Distribution and dynamics of waterbird habitat during spring in southern Oregon–Northeastern California. Western North American Naturalist, 70(1), 26–38. 10.3398/064.070.0104

[gcb15010-bib-0032] Foga, S. , Scaramuzza, P. L. , Guo, S. , Zhu, Z. , Dilley, R. D. , Beckmann, T. , … Laue, B. (2017). Cloud detection algorithm comparison and validation for operational Landsat data products. Remote Sensing of Environment, 194, 379–390. 10.1016/j.rse.2017.03.026

[gcb15010-bib-0033] Frazier, S. (1999). Ramsar sites overview: A synopsis of the world's wetlands of international importance. Wageningen, Netherlands: Wetlands International, 48 pp. Retrieved from https://www.cabdirect.org/cabdirect/abstract/20036792915

[gcb15010-bib-0034] Fritze, H. , Stewart, I. T. , & Pebesma, E. (2011). Shifts in Western North American snowmelt runoff regimes for the recent warm decades. Journal of Hydrometeorology, 12(5), 989–1006. 10.1175/2011jhm1360.1

[gcb15010-bib-0035] Fyfe, J. C. , Derksen, C. , Mudryk, L. , Flato, G. M. , Santer, B. D. , Swart, N. C. , … Jiao, Y. (2017). Large near‐term projected snowpack loss over the western United States. Nature Communications, 8(1), 1–7. 10.1038/ncomms14996 PMC5399290 28418406

[gcb15010-bib-0036] Getches, D. H. , Zellmer, S. B. , & Amos, A. L. (2009). Water law in a nutshell (4th ed .). St. Paul, MN: West Publishing Company, 492 pp.

[gcb15010-bib-0037] Gilbert, N. (2011). United Nations considers creating advisory panel on land degradation akin to IPCC. Nature, 477, 262–264. 10.1038/477262a 21921893

[gcb15010-bib-0038] González‐Gajardo, A. , Sepúlveda, P. V. , & Schlatter, R. (2009). Waterbird assemblages and habitat characteristics in wetlands: Influence of temporal variability on species‐habitat relationships. Waterbirds, 32(2), 225–233. 10.1675/063.032.0203

[gcb15010-bib-0039] Gorelick, N. , Hancher, M. , Dixon, M. , Ilyushchenko, S. , Thau, D. , & Moore, R. (2017). Google Earth Engine: Planetary‐scale geospatial analysis for everyone. Remote Sensing of Environment, 202, 18–27. 10.1016/j.rse.2017.06.031

[gcb15010-bib-0040] Grafton, R. Q. , Williams, J. , Perry, C. J. , Molle, F. , Ringler, C. , Steduto, P. , … Allen, R. G. (2018). The paradox of irrigation efficiency. Science, 361(6404), 748–750. 10.1126/science.aat9314 30139857

[gcb15010-bib-0041] Haig, S. M. , Mehlman, D. W. , & Oring, L. W. (1998). Avian movements and wetland connectivity in landscape conservation. Conservation Biology, 12(4), 749–758. 10.1111/j.1523-1739.1998.97102.x

[gcb15010-bib-0042] Haig, S. M. , Murphy, S. P. , Matthews, J. H. , Arismendi, I. , & Safeeq, M. (2019). Climate‐altered wetlands challenge waterbird use and migratory connectivity in arid landscapes. Scientific Reports, 9, 1–10. 10.1038/s41598-019-41135-y 30874622 PMC6420639

[gcb15010-bib-0043] Halabisky, M. , Moskal, L. M. , Gillespie, A. , & Hannam, M. (2016). Reconstructing semi‐arid wetland surface water dynamics through spectral mixture analysis of a time series of Landsat satellite images (1984–2011). Remote Sensing of Environment, 177, 171–183. 10.1016/j.rse.2016.02.040

[gcb15010-bib-0044] Hassanli, A. M. , Ebrahimizadeh, M. A. , & Beecham, S. (2009). The effects of irrigation methods with effluent and irrigation scheduling on water use efficiency and corn yields in an arid region. Agricultural Water Management, 96(1), 93–99. 10.1016/j.agwat.2008.07.004

[gcb15010-bib-0045] Herbst, D. B. (2006). Salinity controls on trophic interactions among invertebrates and algae of solar evaporation ponds in the Mojave Desert and relation to shorebird foraging and selenium risk. Wetlands, 26(2), 475–485. 10.1672/0277-5212(2006)26[475:scotia]2.0.co;2

[gcb15010-bib-0046] Hua, N. , Tan, K. , Chen, Y. , & Ma, Z. (2015). Key research issues concerning the conservation of migratory shorebirds in the Yellow Sea region. Bird Conservation International, 25(1), 38–52. 10.1017/S0959270914000380

[gcb15010-bib-0047] Hughes, S. , Pincetl, S. , & Boone, C. (2013). Triple exposure: Regulatory, climatic, and political drivers of water management changes in the city of Los Angeles. Cities, 32, 51–59. 10.1016/j.cities.2013.02.007

[gcb15010-bib-0048] Intermountain West Joint Venture (IWJV) . (2013). Intermountain West Joint Venture. 2013 Implementation plan—Strengthening science and partnerships. Intermountain West Joint Venture, 379 pp. Retrieved from https://iwjv.org/resource/iwjv-2013-implementation-plan-entire-plan/

[gcb15010-bib-0049] Ishwaran, H. , & Kogalur, U. B. (2019). Fast unified random forests for survival, regression, and classification (RF-SRC). R package version, 2(1). Retrieved from https://cran.r-project.org/package=randomForestSRC

[gcb15010-bib-0050] Ishwaran, H. , & Lu, M. (2019). Standard errors and confidence intervals for variable importance in random forest regression, classification, and survival. Statistics in Medicine, 38(4), 558–582. 10.1002/sim.7803 29869423 PMC6279615

[gcb15010-bib-0051] Jehl, J. R. Jr. (1994). Changes in saline and alkaline lake avifaunas in western North America in the past 150 years. Studies in Avian Biology, 15, 258–272. Retrieved from https://sora.unm.edu/node/139484.

[gcb15010-bib-0052] Jin, H. H. , Lang, C. , Yeo, M. W. , Stehman, I.‐Y. , & Stephen, V. (2017). Monitoring of wetland inundation dynamics in the Delmarva Peninsula using Landsat time‐series imagery from 1985 to 2011. Remote Sensing of Environment, 190, 26–41. 10.1016/j.rse.2016.12.001

[gcb15010-bib-0053] Jolly, I. D. , McEwan, K. L. , & Holland, K. L. (2008). A review of groundwater–surface water interactions in arid/semi‐arid wetlands and the consequences of salinity for wetland ecology. Ecohydrology, 1(1), 43–58. 10.1002/eco.6

[gcb15010-bib-0054] Kendy, E. , Aylward, B. , Ziemer, L. S. , Richter, B. D. , Colby, B. G. , Grantham, T. E. , … Kappel, C. V. (2018). Water transactions for streamflow restoration, water supply reliability, and rural economic vitality in the Western United States. Journal of the American Water Resources Association, 54(2), 487–504. 10.1111/1752-1688.12619

[gcb15010-bib-0055] Kirby, J. S. , Stattersfield, A. J. , Butchart, S. H. M. , Evans, M. I. , Grimmett, R. F. A. , Jones, V. R. , … Newton, I. (2008). Key conservation issues for migratory land‐ and waterbird species on the world's major flyways. Bird Conservation International, 18(S1), S49–S73. 10.1017/S0959270908000439

[gcb15010-bib-0056] Klos, P. Z. , Link, T. E. , & Abatzoglou, J. T. (2014). Extent of the rain‐snow transition zone in the western US under historic and projected climate. Geophysical Research Letters, 41(13), 4560–4568. 10.1002/2014GL060500

[gcb15010-bib-0057] Koons, D. N. , Gunnarsson, G. , Schmutz, J. M. , & Rotella, J. J. (2014). Drivers of waterfowl population dynamics: From teal to swans. Wildfowl, 169–191. Retrieved from https://wildfowl.wwt.org.uk/index.php/wildfowl/article/viewFile/2606/1724

[gcb15010-bib-0058] Kushlan, J. A. , Steinkamp, M. J. , Parsons, K. C. , Capp, J. , Cruz, M. A. , Coulter, M. , … Elliot, R. (2002). Waterbird conservation for the Americas: The North American waterbird conservation plan, version 1. U.S. Fish and Wildlife Service, 78 pp. Retrieved from https://pubs.er.usgs.gov/publication/5200307

[gcb15010-bib-0059] Larson, R. , Eilers, J. , Kreuz, K. , Pecher, W. T. , DasSarma, S. , & Dougill, S. (2016). Recent desiccation‐related ecosystem changes at Lake Abert, Oregon: A terminal alkaline salt lake. Western North American Naturalist, 76(4), 389–404. 10.3398/064.076.0402

[gcb15010-bib-0060] Lehner, B. , & Grill, G. (2013). Global river hydrography and network routing: Baseline data and new approaches to study the world's large river systems. Hydrological Processes, 27(15), 2171–2186. 10.1002/hyp.9740

[gcb15010-bib-0061] Lincoln, F. (1935). The migratory flyways of North America. U.S. Department of Agriculture. Circular, 342, 12 Retrieved from https://www.biodiversitylibrary.org/item/130083#page/7/mode/1up.

[gcb15010-bib-0062] Masek, J. G. , Vermote, E. F. , Saleous, N. E. , Wolfe, R. , Hall, F. G. , Huemmrich, K. F. , … Lim, T.‐K. (2006). A Landsat surface reflectance dataset for North America, 1990–2000. IEEE Geoscience and Remote Sensing Letters, 3(1), 68–72. 10.1109/LGRS.2005.857030

[gcb15010-bib-0063] Mason, I. M. , Guzkowska, M. A. J. , Rapley, C. G. , & Street‐Perrott, F. A. (1994). The response of lake levels and areas to climatic change. Climatic Change, 27(2), 161–197. 10.1007/BF01093590

[gcb15010-bib-0064] Maupin, M. A. , Kenny, J. F. , Hutson, S. S. , Lovelace, O. K. , Barber, N. L. , & Linsey, K. S. (2010). USGS circular 1405: Estimated use of water in the United States in 2010. U.S. Geological Survey, 55 10.3133/cir1405

[gcb15010-bib-0065] McBeth, D. (1997). Wetlands Conservation and Federal Regulation: Analysis of the Food Security Act's Swampbuster Provisions as Amended by the Federal Agriculture Improvement and Reform Act of 1996. Harvard Environmental Law Review, 21, 201 Retrieved from https://heinonline.org/hol-cgi-bin/get_pdf.cgi?handle=hein.journals/helr21%26section=9

[gcb15010-bib-0066] McFarland, W. D. , & Ryals, G. N. (1991). Adequacy of available hydrogeologic data for evaluation of declining ground‐water levels in the Fort Rock Basin, south‐central Oregon. Water‐Resources Investigations Report, 89, 4057 Retrieved from https://pubs.usgs.gov/wri/1989/4057/report.pdf

[gcb15010-bib-0067] McFeeters, S. K. (1996). The use of the Normalized Difference Water Index (NDWI) in the delineation of open water features. International Journal of Remote Sensing, 17(7), 1425–1432. 10.1080/01431169608948714

[gcb15010-bib-0068] McWethy, D. B. , & Austin, J. E. (2009). Nesting ecology of greater sandhill cranes (*Grus canadensis tabida*) in riparian and palustrine wetlands of Eastern Idaho. Waterbirds, 32(1), 106–115. 10.1675/063.032.0112

[gcb15010-bib-0069] Meier, J. , Zabel, F. , & Mauser, W. (2018). A global approach to estimate irrigated areas—A comparison between different data and statistics. Hydrology and Earth System Sciences, 22(2), 1119–1133. 10.5194/hess-22-1119-2018

[gcb15010-bib-0070] Melchiorri, M. , & Siragusa, A. (2018). Analyzing cities with the global human settlement layer: A methodology to compare urban growth using remote sensing data In Bisello A., Vettorato D., Laconte P., & Costa S. (Eds.), Smart and sustainable planning for cities and regions (pp. 151–165). New York, NY: Springer International Publishing.

[gcb15010-bib-0071] Mifflin, M. D. , & Wheat, M. M. (1979). Pluvial lakes and estimated pluvial climates of Nevada. University of Nevada Reno, 60 pp. Retrieved from https://www.nrc.gov/docs/ML0333/ML033350348.pdf

[gcb15010-bib-0072] Moore, J. N. (2016). Recent desiccation of Western Great Basin saline lakes: Lessons from Lake Abert, Oregon, USA. The Science of the Total Environment, 554, 142–154. 10.1016/j.scitotenv.2016.02.161 26950628

[gcb15010-bib-0073] Morrison, R. I. G. , & Myers, J. P. (1989). Shorebird flyways in the new world In Boyd H. & Pirot J. Y. (Eds.), Flyways and reserve networks for water birds (pp. 85–96). London, UK: International Waterfowl and Wetlands Research Bureau.

[gcb15010-bib-0074] Moulton, C. , Carlisle, J. , Brenner, K. , & Cavallaro, R. (2013). Assessment of foraging habitats of white‐faced Ibis near two important breeding colonies in Eastern Idaho. Boise, ID: Idaho Fish and Game, 30 pp. Retrieved from https://www.semanticscholar.org/paper/Assessment-of-Foraging-Habitats-of-White-faced-Ibis-Moulton-Carlisle/648ef769227a44868256da25090b7ea6524a7656

[gcb15010-bib-0075] Murray, N. J. , Marra, P. P. , Fuller, R. A. , Clemens, R. S. , Dhanjal‐Adams, K. , Gosbell, K. B. , … Studds, C. E. (2018). The large‐scale drivers of population declines in a long‐distance migratory shorebird. Ecography, 41(6), 867–876. 10.1111/ecog.02957

[gcb15010-bib-0076] NAWMP , Canadian Wildlife Service , U.S. Fish and Wildlife Service & Ambiente y Recursos Naturales . (2012). North American waterfowl management plan: People conserving waterfowl and wetlands. 48 pp. Retrieved from https://www.fws.gov/migratorybirds/pdf/management/NAWMP/2012NAWMP.pdf

[gcb15010-bib-0077] *North American Wetlands Conservation Act* . (1989). P.L. 101‐233.

[gcb15010-bib-0078] Oring, L. W. , Neel, L. , & Oring, K. E. (2000). Intermountain west regional shorebird plan. Intermountain West Joint Venture, 55 pp. Retrieved from https://www.shorebirdplan.org/wp-content/uploads/2013/01/IMWEST4.pdf

[gcb15010-bib-0079] Oring, L. W. , & Reed, J. (1997). Shorebirds of the western Great Basin of North America: Overview and importance to continental populations. International Wader Studies, 9, 6–12. Retrieved from https://sora.unm.edu/sites/default/files/journals/iws/n009/p00006-p00014.pdf

[gcb15010-bib-0080] Paul, D. S. , & Manning, A. E. (2002). Great Salt Lake waterbird survey five‐year report (1997–2001). Great Salt Lake Ecosystem Program and Utah Division of Wildlife Resources, 64 pp. Retrieved from https://wildlife.utah.gov/gsl/waterbirdsurvey/

[gcb15010-bib-0081] Peck, D. E. , McLeod, D. M. , Hewlett, J. P. , & Lovvorn, J. R. (2004). Irrigation‐dependent wetlands versus instream flow enhancement: Economics of water transfers from agriculture to wildlife uses. Environmental Management, 34(6), 842–855. 10.1007/s00267-004-3085-z 15633027

[gcb15010-bib-0082] Pérez‐Arteaga, A. , Gaston, K. J. , & Kershaw, M. (2002). Undesignated sites in Mexico qualifying as wetlands of international importance. Biological Conservation, 107(1), 47–57. 10.1016/S0006-3207(02)00043-5

[gcb15010-bib-0083] Pettorelli, N. , Vik, J. O. , Mysterud, A. , Gaillard, J.‐M. , Tucker, C. J. , & Stenseth, N. C. (2005). Using the satellite‐derived NDVI to assess ecological responses to environmental change. Trends in Ecology & Evolution, 20(9), 503–510. 10.1016/j.tree.2005.05.011 16701427

[gcb15010-bib-0084] Pfeiffer, L. , & Lin, C.‐Y.‐C. (2014). Does efficient irrigation technology lead to reduced groundwater extraction? Empirical evidence. Journal of Environmental Economics and Management, 67(2), 189–208. 10.1016/j.jeem.2013.12.002

[gcb15010-bib-0085] Pool, D. B. , Panjabi, A. O. , Macias‐Duarte, A. , & Solhjem, D. M. (2014). Rapid expansion of croplands in Chihuahua, Mexico threatens declining North American grassland bird species. Biological Conservation, 170, 274–281. 10.1016/j.biocon.2013.12.019

[gcb15010-bib-0086] Pritchett, D. , & Manning, S. J. (2012). Response of an intermountain groundwater‐dependent ecosystem to water table drawdown. Western North American Naturalist, 72(1), 48–59. 10.3398/064.072.0106

[gcb15010-bib-0087] QGIS Development Team . (2017). QGIS geographic information system. Open Source Geospatial Foundation Retrieved from http://qgis.org

[gcb15010-bib-0088] R Core Team . (2018). R: A language and environment for statistical computing. Vienna, Austria: R Foundation for Statistical Computing Retrieved from https://www.R-project.org

[gcb15010-bib-0089] Rakhimberdiev, E. , Duijns, S. , Karagicheva, J. , Camphuysen, C. J. , Dekinga, A. , Dekker, R. , … Piersma, T. (2018). Fuelling conditions at staging sites can mitigate Arctic warming effects in a migratory bird. Nature Communications, 9(1), 4263 10.1038/s41467-018-06673-5 PMC6189115 30323300

[gcb15010-bib-0090] Richter, B. D. , Brown, J. D. , DiBenedetto, R. , Gorsky, A. , Keenan, E. , Madray, C. , … Ryu, S. (2017). Opportunities for saving and reallocating agricultural water to alleviate water scarcity. Water Policy, 19(5), 886–907. 10.2166/wp.2017.143

[gcb15010-bib-0091] Riojas‐López, M. E. , & Mellink, E. (2005). Potential for biological conservation in man‐modified semiarid habitats in northeastern Jalisco, Mexico. Biodiversity and Conservation, 14(9), 2251–2263. 10.1007/s10531-004-5289-1

[gcb15010-bib-0092] Rocha, A. R. , Silva, R. , Villegas, A. , Sánchez‐Guzmán, J. M. , Ramos, J. A. , & Masero, J. A. (2016). Physiological, morphological and behavioural responses of self‐feeding precocial chicks copying with contrasting levels of water salinity during development. PLoS ONE, 11(10), e0165364 10.1371/journal.pone.0165364 27788200 PMC5082863

[gcb15010-bib-0093] Roshier, D. A. , Robertson, A. I. , Kingsford, R. T. , & Green, D. G. (2001). Continental‐scale interactions with temporary resources may explain the paradox of large populations of desert waterbirds in Australia. Landscape Ecology, 16(6), 547–556. 10.1023/A:1013184512541

[gcb15010-bib-0094] RStudio Team . (2016). RStudio: Integrated Development Environment for R. Boston, MA: RStudio Inc Retrieved from http://www.rstudio.com/

[gcb15010-bib-0095] Scott, C. A. , Vicuña, S. , Blanco, G. I. , Meza, F. , & Varela, O. C. (2014). Irrigation efficiency and water‐policy implications for river‐basin resilience. Hydrology and Earth System Sciences, 18(4), 1339–1348. 10.5194/hess-18-1339-2014

[gcb15010-bib-0096] Seddon, A. W. R. , Macias‐Fauria, M. , Long, P. R. , Benz, D. , & Willis, K. J. (2016). Sensitivity of global terrestrial ecosystems to climate variability. Nature, 531(7593), 229–232. 10.1038/nature16986 26886790

[gcb15010-bib-0097] Sedinger, J. S. , & Alisauskas, R. T. (2014).Cross‐seasonal effects and the dynamics of waterfowl populations. *Wildfowl; 2014: Wildfowl Special Issue No. 4 277–304* Retrieved from http://wildfowl.wwt.org.uk/index.php/wildfowl/article/view/2609

[gcb15010-bib-0098] Senner, N. R. , Moore, J. N. , Seager, S. T. , Dougill, S. , Kreuz, K. , & Senner, S. E. (2018). A salt lake under stress: Relationships among birds, water levels, and invertebrates at a Great Basin saline lake. Biological Conservation, 220, 320–329. 10.1016/j.biocon.2018.02.003

[gcb15010-bib-0099] Senner, S. E. , Andres, B. A. , & Gates, H. R. (2016). Pacific Americas shorebird conservation strategy The National Audubon Society, 81 pp. Retrieved from https://www.shorebirdplan.org/wp-content/uploads/2017/03/Pacific-Americas-Strategy-2016.pdf

[gcb15010-bib-0100] Siegel, S. (1957). Nonparametric statistics. The American Statistician, 11(3), 13–19. 10.1080/00031305.1957.10501091

[gcb15010-bib-0101] Skagen, S. K. , Granfors, D. A. , & Melcher, C. P. (2008). On determining the significance of ephemeral continental wetlands to North American migratory shorebirds. The Auk, 125(1), 20–29. 10.1525/auk.2008.125.1.20

[gcb15010-bib-0102] Small‐Lorenz, S. L. , Culp, L. A. , Ryder, T. B. , Will, T. C. , & Marra, P. P. (2013). A blind spot in climate change vulnerability assessments. Nature Climate Change, 3, 91 10.1038/nclimate1810

[gcb15010-bib-0103] Szeptycki, L. F. , Forgie, J. , Hook, E. , Lorick, K. , & Womble, P. (2015). Environmental water rights transfers: A review of state laws. Utah State University, Libraries Retrieved from https://digitalcommons.usu.edu/instream_all/3/

[gcb15010-bib-0104] Thomas, J. M. (1995). Water budget and salinity of Walker Lake, western Nevada. US Geological Survey, 2 pp. Retrieved from https://pubs.er.usgs.gov/publication/fs11595

[gcb15010-bib-0105] Vélez, E. P. , & Saez, E. M. (2011). Water use for agriculture in Mexico In Oswald Spring O. (Ed.), Water resources in Mexico: Scarcity, degradation, stress, conflicts, management, and policy (pp. 129–143). Berlin, Heidelberg, Germany: Springer.

[gcb15010-bib-0106] Verduzco, V. S. , Vivoni, E. R. , Yépez, E. A. , Rodríguez, J. C. , Watts, C. J. , Tarin, T. , … Ivanov, V. Y. (2018). Climate change impacts on net ecosystem productivity in a subtropical shrubland of Northwestern México. Journal of Geophysical Research: Biogeosciences, 123(2), 688–711. 10.1002/2017JG004361

[gcb15010-bib-0107] Vermote, E. , Justice, C. , Claverie, M. , & Franch, B. (2016). Preliminary analysis of the performance of the Landsat 8/OLI land surface reflectance product. Remote Sensing of Environment, 185, 46–56. 10.1016/j.rse.2016.04.008 32020955 PMC6999666

[gcb15010-bib-0108] Wada, Y. , van Beek, L. P. H. , Viviroli, D. , Dürr, H. H. , Weingartner, R. , & Bierkens, M. F. P. (2011). Global monthly water stress: 2. Water demand and severity of water stress. Water Resources Research, 47(7), 1–17. 10.1029/2010WR009792

[gcb15010-bib-0109] Wada, Y. , van Beek, L. P. H. , Wanders, N. , & Bierkens, M. F. P. (2013). Human water consumption intensifies hydrological drought worldwide. The Environmentalist, 8(3), 14 10.1088/1748-9326/8/3/034036

[gcb15010-bib-0110] Wang, J. , Song, C. , Reager, J. T. , Yao, F. , Famiglietti, J. S. , Sheng, Y. , … Wada, Y. (2018). Recent global decline in endorheic basin water storages. Nature Geoscience, 11, 926–932. 10.1038/s41561-018-0265-7 PMC6267997 30510596

[gcb15010-bib-0111] Ward, F. A. , & Pulido‐Velazquez, M. (2008). Water conservation in irrigation can increase water use. Proceedings of the National Academy of Sciences of the United States of America, 105(47), 18215–18220. 10.1073/pnas.0805554105 19015510 PMC2584147

[gcb15010-bib-0112] Wetlands International . (2012). Waterbird population estimates (5th ed .). Wageningen, Netherlands: Wetlands International, 25 pp. Retrieved from https://www.wetlands.org/wp-content/uploads/2015/11/Waterbird-Populations-Estimates-Fifth-Edition.pdf

[gcb15010-bib-0113] Wickham, H. (2017). Tidyverse R package version 1.2. 1. Vienna, Austria: R Core Team.

[gcb15010-bib-0114] Wilsey, C. B. , Taylor, N. , Stockdale, M. , & Stockdale, K. (2017). Water and birds in the Arid West: Habitats in decline. National Audubon Society, 51 pp. Retrieved from https://www.audubon.org/news/executive-summary-water-and-birds-arid-west-habitats-decline

[gcb15010-bib-0115] Wollheim, W. M. , & Lovvorn, J. R. (1995). Salinity effects on macroinvertebrate assemblages and waterbird food webs in shallow lakes of the Wyoming high plains. Hydrobiologia, 310(3), 207–233. 10.1007/BF00006832

[gcb15010-bib-0116] Wurtsbaugh, W. A. , Miller, C. , Null, S. E. , DeRose, R. J. , Wilcock, P. , Hahnenberger, M. , … Moore, J. (2017). Decline of the world's saline lakes. Nature Geoscience, 10, 816 10.1038/ngeo3052

[gcb15010-bib-0117] Xu, Y. , Si, Y. , Wang, Y. , Zhang, Y. , Prins, H. H. T. , Cao, L. , & de Boer, W. F. (2019). Loss of functional connectivity in migration networks induces population decline in migratory birds. Ecological Applications, 29(7), e01960 10.1002/eap.1960 31237968 PMC6852588

[gcb15010-bib-0118] Zanella, L. , Folkard, A. M. , Blackburn, G. A. , & Carvalho, L. M. T. (2017). How well does random forest analysis model deforestation and forest fragmentation in the Brazilian Atlantic forest? Environmental and Ecological Statistics, 24(4), 529–549. 10.1007/s10651-017-0389-8

[gcb15010-bib-0119] Zhang, G. , & Lu, Y. (2012). Bias‐corrected random forests in regression. Journal of Applied Statistics, 39(1), 151–160. 10.1080/02664763.2011.578621

