# Supplementary material for: Climate and human water use diminish wetland networks supporting continental waterbird migration
Source: Glob Chang Biol. 2020 Feb 13;26(4):2042–59. doi: 10.1111/gcb.15010 (PMC7155039; doi:10.1111/gcb.15010)
Supplement: Supplementary file 1 [file GCB-26-2042-s001.docx]

**Supporting Information Figures S1—S16**

**Figures S1-S9**

Boxplots of annual (1984-2018) lake and wetland surface water area and human-water-use and climate variables. Human water use variables are represented by irrigated agricultural extent and human population density (people/sq-km, PopDen). Climate variables are: runoff (*RO*), evapotranspiration (*ET*), precipitation (*PR*); and snow water equivalent (*SWE*). Variables are summarized for two monitoring periods P1 (1984-1999) and P2 (2000-2018). See methods for data sources and details of analyses.

Boxes in all boxplots encompasses the 25^th^ to 75^th^ percentiles of the data. The heavy line dividing the box horizontally is the median value. The whiskers are the 5^th^ and 95^th^ percentiles of the distribution, with “outliers” represented as dots.

*P*-values for examining the difference between periods were calculated using a non-parametric Wilcoxon test. The null hypothesis of the Wilcoxon test is that true location shift (median shift) is equal to zero. The alternative hypothesis is that location shift is not equal to zero. Rejecting the null hypothesis (low *P*-value, e.g., *P*≤0.05) means we have evidence that the medians of the two factors differ. Accepting the null hypothesis (high *P*-value, e.g., *P*>0.05) suggests that the two factors have the same distribution with the same median.

Basin locations are in Figure 1 of text.

Snowmelt

Monsoon

Figure S1. Boxplots of Lake area for snowmelt and monsoonal watersheds between monitoring periods (P1: 1984-1999; P2: 2000-2018). *P*-values are from Wilcoxon test.

Snowmelt

Monsoon

Figure S2. Boxplots of wetland area for snowmelt and monsoonal watersheds between monitoring periods (P1: 1984-1999; P2: 2000-2018). *P*-values are from Wilcoxon test.

Snowmelt

Monsoon

Figure S3. Boxplots of irrigated agricultural extent for snowmelt and monsoonal watersheds between monitoring periods (P1: 1984-1999; P2: 2000-2018). *P*-values are from Wilcoxon test.

Snowmelt

Monsoon

Figure S4. Boxplots of human population density (number/km^2^) for snowmelt and monsoonal watersheds between monitoring periods (P1: 1984-1999; P2: 2000-2018). *P*-values are from Wilcoxon test.

Snowmelt

Monsoon

Figure S5. Boxplots of evapotranspiration (ET, mm) for snowmelt and monsoonal watersheds between monitoring periods (P1: 1984-1999; P2: 2000-2018). *P*-values are from Wilcoxon test.

Snowmelt

Monsoon

Figure S6. Boxplots of precipitation (PR, mm) for snowmelt and monsoonal watersheds between monitoring periods (P1: 1984-1999; P2: 2000-2018). *P*-values are from Wilcoxon test.

Snowmelt

Monsoon

Figure S7. Boxplots of runoff (RO, mm) for snowmelt and monsoonal watersheds between monitoring periods (P1: 1984-1999; P2: 2000-2018). *P*-values are from Wilcoxon test.

Snowmelt

Monsoon

Figure S8. Boxplots of snow water equivalent (SWE, mm) for snowmelt and monsoonal watersheds between monitoring periods (P1: 1984-1999; P2: 2000-2018). *P*-values are from Wilcoxon test.

**Figures S9-S16**

Time series plots of annual (1984-2018) lake and wetland surface water area and human-water-use and climate variables. Human water use variables are represented by irrigated agricultural extent and human population density (people/sq-km, PopDen). Climate variables are: runoff (*RO*), evapotranspiration (*ET*), precipitation (*PR*); and snow water equivalent (*SWE*).

See Methods in the text for data origin and details of analyses.

Curved black lines within plots represent annual change to variables and straight line the least-squares best fit with 95% CI for the slope in grey. Least-squares are shown as an indicator of trend not as a linear model of change because many linear fits account for small amounts of the variance and have irregular distributions for residuals. Instead we use the boxplots in the previous section to examine statistical difference between, and hence change, between periods (see text and Figures S1-S9).

Basin locations are in Figure 1 of the text.

Snowmelt

Monsoon

Figure S9. Annual (1984-2018) endorheic lakes surface water trends for snowmelt and monsoonal watersheds. Straight line is least-squares best fit with 95% CI for the slope in grey.

Snowmelt

Monsoon

Figure S10. Annual (1984-2018) wetland area trends for snowmelt and monsoonal watersheds. Straight line is least-squares best fit with 95% CI for the slope in grey.

Snowmelt

Monsoon

Figure S11. Annual (1984-2018) irrigated agricultural extent for snowmelt and monsoonal watersheds. Straight line is least-squares best fit with 95% CI for the slope in grey.

Snowmelt

Monsoon

Figure S12. Annual (1974-2018) human population density for snowmelt and monsoonal watersheds. Straight line is least-squares best fit with 95% CI for the slope in grey.

Snowmelt

Monsoon

Figure S13. Annual (1983-2018) evapotranspiration (ET, mm) for snowmelt and monsoonal watersheds. Straight line is least-squares best fit with 95% CI for the slope in grey.

Snowmelt

Monsoon

Figure S14. Annual (1983-2018) precipitation (PR, mm) for snowmelt and monsoonal watersheds. Straight line is least-squares best fit with 95% CI for the slope in grey.

Snowmelt

Monsoon

Figure S15. Annual (1983-2018) runoff (RO, mm) for snowmelt and monsoonal watersheds. Straight line is least-squares best fit with 95% CI for the slope in grey.

Snowmelt

Monsoon

Figure S16. Annual (1983-2018) snow water equivalent (SWE, mm) for snowmelt and monsoonal watersheds. Straight line is least-squares best fit with 95% CI for the slope in grey.

**Supporting Information Tables S1-S10**

Locations of watersheds are in text Figure 1.

Boxplots and line graphs of all data are in the Supporting Information figures.

Table S1. Endorheic lake area changes between periods P1 (1984-99) and P2 (2000-18) and statistical test results of significance from the Wilcoxon test and linear regression on the annual time series.

Table S2. Endorheic wetland area changes between periods P1 (1984-99) and P2 (2000-18) and statistical test results of significance from the Wilcoxon test and linear regression on the annual time series.

Table S3. Endorheic peripheral wetland area changes changes between periods P1 (1984-99) and P2 (2000-18).

Table S4. Irrigated agricultural extent changes between periods P1 (1984-99) and P2 (2000-18) and statistical test results of significance from the Wilcoxon test and linear regression on the annual time series.

Table S5. Population density changes between periods P1 (1984-99) and P2 (2000-18) and statistical test results of significance from the Wilcoxon test and linear regression on the annual time series.

Table S6. Evapotranspiration (ET) changes between periods P1 (1984-99) and P2 (2000-18) and statistical test results of significance from the Wilcoxon test and linear regression on the annual time series.

Table S7. Precipitation (PR) changes between periods P1 (1984-99) and P2 (2000-18) and statistical test results of significance from the Wilcoxon test and linear regression on the annual time series.

Table S8. Runoff (RO) changes between periods P1 (1984-99) and P2 (2000-18) and statistical test results of significance from the Wilcoxon test and linear regression on the annual time series.

Table S9. Snow water equivalent (SWE) changes between periods P1 (1984-99) and P2 (2000-18) and statistical test results of significance from the Wilcoxon test and linear regression on the annual time series.

Table S10. Averaged proportion of natural versus agricultural associated wetland flooding in snowmelt and monsoon watersheds from 1984-2018. Included is the proportion of irrigated agriculture associated with wetland flooding.
